# Revisiting the polytopal rearrangements in penta-coordinate d^7^-metallocomplexes: modified Berry pseudorotation, octahedral switch, and butterfly isomerization†
†Electronic supplementary information (ESI) available. See DOI: 10.1039/c7sc00703e



**DOI:** 10.1039/c7sc00703e

**Published:** 2017-06-02

**Authors:** Rubik Asatryan, Eli Ruckenstein, Johannes Hachmann

**Affiliations:** a Department of Chemical and Biological Engineering , University at Buffalo , The State University of New York , Buffalo , NY 14260 , USA . Email: rubikasa@buffalo.edu ; Email: hachmann@buffalo.edu; b New York State Center of Excellence in Materials Informatics , Buffalo , NY 14203 , USA; c Computational and Data-Enabled Science and Engineering Graduate Program , University at Buffalo , The State University of New York , Buffalo , NY 14260 , USA

## Abstract

This paper provides a first-principles theoretical investigation of the polytopal rearrangements and fluxional behavior of five-coordinate d^7^-transition metal complexes. Our work is primarily based on a potential energy surface analysis of the iron tetracarbonyl hydride radical HFe˙(CO)_4_. We demonstrate the existence of distorted coordination geometries in this prototypical system and, for the first time, introduce three general rearrangement mechanisms, which account for the non-ideal coordination. The first of these mechanisms constitutes a modified version of the Berry pseudorotation *via* a square-based pyramidal *C*_4v_ transition state that connects two chemically identical edge-bridged tetrahedral stereoisomers of *C*_2v_ symmetry. It differs from the classical Berry mechanism, which involves two regular *D*_3h_ equilibrium structures and a *C*_4v_ transition state. The second mechanism is related to the famous “tetrahedral jump” hypothesis, postulated by Muetterties for a number of d^6^ HML_4_ and H_2_ML_4_ complexes. Here, our study suggests two fluxional rearrangement pathways *via* distinct types of *C*_2v_ transition states. Both pathways of this mechanism can be described as a single-ligand migration to a vacant position of an “octahedron”, thus interchanging (switching) the apical and basal ligands of the initial quasi-square pyramidal isomer, which is considered as an idealized octahedron with a vacancy. Accordingly, we call this mechanism “octahedral switch”. The third mechanism follows a butterfly-type isomerization featuring a key-angle deformation, and we thus call it “butterfly isomerization”. It connects the quasi-square pyramidal and edge-bridged tetrahedral isomers of HFe˙(CO)_4_ through a distorted edge-bridged tetrahedral transition state of *C*_s_ symmetry. Our paper discusses the overall features of the isomers and rearrangement mechanisms as well as their implications. We rationalize the existence of each stationary point through an electronic structure analysis and argue their relevance for isolobal analogues of HFe˙(CO)_4_.

## Abstract 2

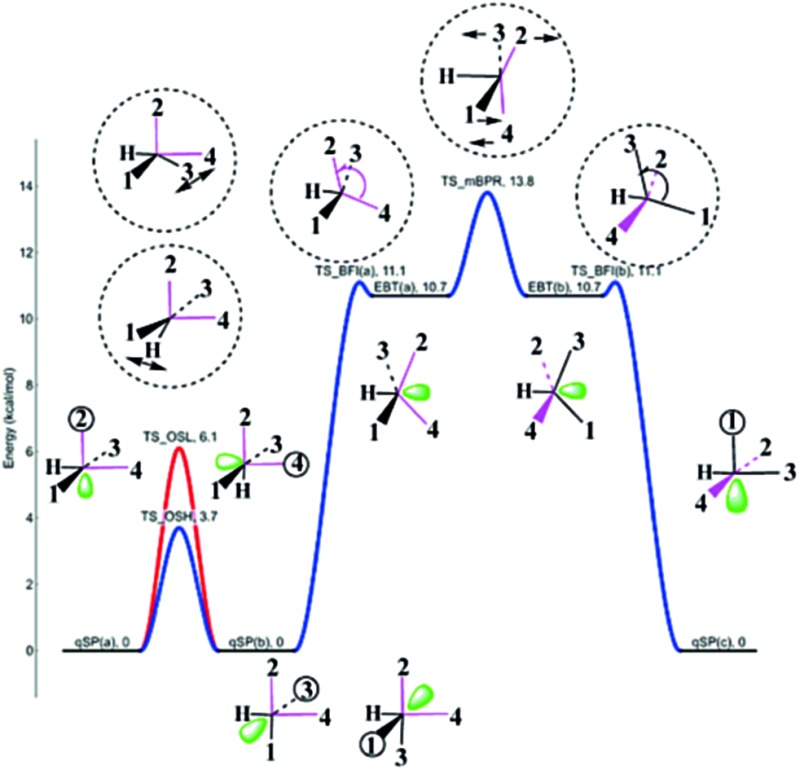
New rearrangement mechanism prototypes are introduced to account for the non-ideal coordination and fluxional behavior of five-coordinate metalo-complexes.

## 1. Introduction

Polytopal rearrangements are chemical transformations that change the ligand positions in the vertices of coordination polyhedra, *i.e.*, they are isomerizations that interconvert different or equivalent spatial arrangements of ligands about a central atom.^1^ The term fluxional rearrangement is often used to indicate the latter case, *i.e.*, the interconversion of chemically identical stereoisomers.^2,3^

Polytopal rearrangements typically involve a variety of intramolecular isomerization, pseudorotation, and other fluxional processes that primarily concern bond-angle alterations, but no ligand elimination or addition.^2–12^ Polytopal rearrangements thus require much lower activation energies than transformations that are associated with the breaking or forming of bonds.^13^ The resulting conformational flexibility affects the (stereo-)selectivity and mechanism of reactions and it thus plays a key role in the stability, reactivity, and catalytic activity of both metallocomplexes and solid state surfaces.^2–6,9–11,13–15^ The structural theory of polytopal processes has been developed on a conceptual level using the valence shell electron pair repulsion (VSEPR) theory^16^ and related points-on-a-sphere models.^9,10,17,18^ A more quantitative, first-principles understanding has been afforded by computational quantum chemistry.^19–25^

Penta-coordinated compounds are of particular interest due to the low-barrier interconversions between the two ideal (*i.e.*, trigonal bipyramidal (TBP) and square pyramidal (SP)) polyhedra as the limiting polytopal structures, which result in an unusually high occurrence of non-standard geometries,^27–33^ as well as their stereochemical non-rigidity (fluxionality).^1–11,19–26^ This situation also gives rise to challenging questions regarding the mechanisms that facilitate these rearrangements.

**Fig. 1:**
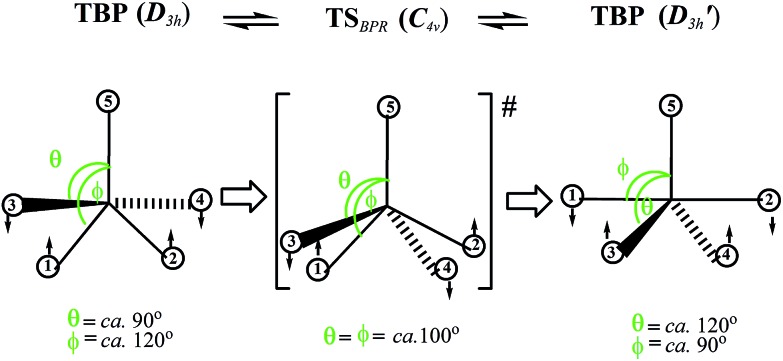
Classical Berry pseudorotation (BPR) mechanism interconverting two trigonal bipyramidal (TBP) stereoisomers of *D*_3h_ symmetry *via* a transition state (TS) of *C*_4v_ symmetry.

A host of hypothetical pathways has been proposed over the past decades for different metal and ligand combinations.^2–11,13a,14,16–43^ For the isomerization in TBP complexes, Muetterties and co-workers have outlined six general mechanisms following topological considerations based on nuclear magnetic resonance (NMR) data.^26^ Of these, the Berry pseudorotation (BPR; see Fig. 1) – the perhaps most prominent intramolecular exchange mechanism in coordination chemistry^34^ – appears to be the only one that could be confirmed based on its ability to explain the experimentally observed stereochemical non-rigidity of penta-coordinate compounds.^3,6,19,24,26,36–38^ The classical BPR mechanism proceeds through a regular SP transition state (TS) of *C*_4v_ symmetry featuring a large apical-M-basal (droop) angle *θ* (Fig. 1).^14,20–22^ In equilibrium SP structures, *θ* is typically ranging from 105° to 125°.^22^ The TS_BPR_ connects two chemically equivalent TBP stereoisomers of *D*_3h_ symmetry, in which the axial and equatorial ligands as switched. The characteristic stereomutation coordinate shown in Fig. 1 indicates that two basal ligands (**1** and **2**) are moving towards the *trans*-position pivotal ligand **5**, while the other two (**3** and **4**) are simultaneously moving away from it.^26b,34^

**Fig. 2:**
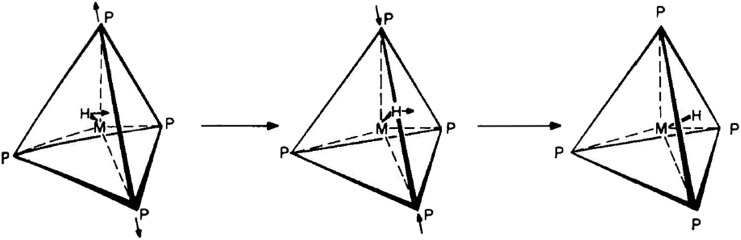
Classical tetrahedral jump (TJ) mechanism that rearranges H-atoms between different facial positions of a quasi-tetrahedron.

Another famous mechanism postulated by Muetterties is the tetrahedral jump (TJ)^3,8,43^ (initially called tetrahedral tunneling^8^) for quasi-tetrahedral HML_4_ and H_2_ML_4_ complexes, in which “hydrogen atoms have been considered to be at tetrahedral facial positions in the ground state”.^43^ To explain their NMR data, the authors proposed a concerted process, in which “a [ligand–metal–ligand] angle increases with concomitant transverse of a hydrogen atom to this affected tetrahedral edge, and then the [ligand–metal–ligand] angle decreases as the H atom goes to a new tetrahedral face”^43^ (see Fig. 2).

It is worth stressing that the TJ mechanism – despite being the subject of an extensive discussion – has never been confirmed by either experiment or calculations.^8,32,59b^

Except for the BPR, the mechanisms outlined in ref. 26 also have never been observed, and the predicted barriers are too high to be accessible under realistic conditions.^24^ A combination of two of these mechanisms constitutes another famous polytopal process, *i.e.*, the turnstile rotation (TSR).^39,40^ It involves an internal rotation of a ligand pair relative to the other three ligands. The TSR interchanges one axial and two equatorial ligands in a motion that resembles that of a turnstile gate. However, the TSR mechanism was dismissed too, in a series of electronic structure studies^24,41,42^ in favor of the BPR. It should be noted, though, that a revised TSR version involving a threefold cyclic permutation (120° rotational motion *vs.* 60° in the original TSR mechanism^39^) was suggested by Lammertsma *et al.* ^24^ A reverse BPR, in which an SP complex rearranges through a TBP TS, has been postulated in early studies as well.^14,44,45^ More recently, the term “reverse BPR” has been used for pathways with edge-bridged (or edge-capped^31^) tetrahedral distortions involving a bending motion of the axial ligands in the opposite direction to the one shown in Fig. 1.^27^ Distortions at different points of the reverse BPR coordinate have been mapped in ref. 31 for a set of experimental structures to complement the data provided by Muetterties for regular BPRs.^46^ In addition, a mini-review by Gusev and Berke^11^ has stressed the importance of two principal types of ligand motion – migratory and replacement – to distinguish the dynamic behavior of metal polyhydrides MH_*n*_L_*m*_ (with *n* ≥ 3). The authors have primarily used this classification to rationalize the fluxional mechanisms in six- and seven-coordinated complexes, while its applicability to five-coordinate systems remains less clear.

The paper at hand addresses the question, which (if any) of the polytopal rearrangement mechanisms suggested in the past are relevant for penta-coordinate (formally) d^7^-systems. Our work offers first-principles mechanistic findings from a careful and comprehensive analysis of the potential energy surfaces (PESs) of a suitable set of coordination compounds. We focus on the iron tetracarbonyl hydride radical HFe˙(CO)_4_ as a simple and clean prototype of this class of systems, and then expand the scope of our discussion to a number of isolobal analogues.

HFe˙(CO)_4_ was first observed by Krusic *et al.* in the mid-1980s using electron paramagnetic resonance (EPR) spectroscopy.^47–49^ Nagorski and Mirbach proposed to utilize this radical as a precursor for the coordinatively unsaturated catalyst HFe˙(CO)_3_ in the hydrogenation of 1-octane.^50^ Our work was motivated by the observation of several elementary reactions involving polytopal rearrangements during a study of radical pathways in the photochemical and thermal hydrogenation of ethylene by Fe(CO)_5_-derived catalysts. (The study of the corresponding molecular pathways was reported in ref. 15.) We found that these rearrangements play an intricate role in the switching between alternative catalytic pathways. A full account of our findings for the [HFe˙(CO)_4_/HFe˙(CO)_3_]-catalyzed hydrogenation of ethylene will be reported elsewhere.

In Section 2 of this paper we will provide a short overview of the computational methodology used in current work. Section 3 describes our investigations of the stationary points found on the HFe˙(CO)_4_ PES. We will analyze the resulting isomers, polytopal rearrangement mechanisms, and overall PES features before putting them into the context of prior work on this topic. We will also rationalize our findings based on electronic structure considerations, subsequently compare the HFe˙(CO)_4_ prototype with other coordination compounds, and discuss the transferability of our findings. We will summarize the insights gained from this work in Section 4.

## 2. Methods and computational details

The study at hand is primarily based on Kohn–Sham density functional theory (DFT)^52,53^ using generalized gradient approximation (GGA)^54^ and hybrid functionals.^55^ In spite of a number of well-known deficiencies and limitations for more complex electronic structure situations, DFT is widely used as a baseline tool in computational coordination chemistry.^56^ Its performance has been extensively studied for various properties and systems, including cases in the same problem domain as the work presented here.^15,36,51,57–59,64^ For instance, the barrier height of the iconic BPR rearrangement in Fe(CO)_5_ was computed by Harris *et al.* at the BP86 GGA level to be 2.1 kcal mol^–1^,^36^ which is in remarkably good agreement to the experimental activation energy of 1.6 ± 0.3 kcal mol^–1^ obtained *via* temperature-dependent two-dimensional infrared spectroscopy.^36^ While no experimental data is available for the geometries of the HFe˙(CO)_4_ isomers to benchmark the employed geometry optimization approach, it is known that DFT successfully predicts the geometries of the precursor H_2_Fe(CO)_4_ (ref. 58 and 59*a*) and various open-shell derivatives of the HFe˙(CO)_4_ radical (a review can be found in ref. 15).

In addition to the DFT study, we also provide higher-level *ab initio* wavefunction theory results to support key findings of our work. These include second-order Møller–Plesset perturbation theory (MP2),^60^ and coupled cluster theory with singles, doubles (CCSD), and perturbative triples amplitudes (CCSD(T)).^61^ The latter is considered the “gold standard” of computational quantum chemistry (see, *e.g.*, ref. 15, 62–64).

**Fig. 3:**
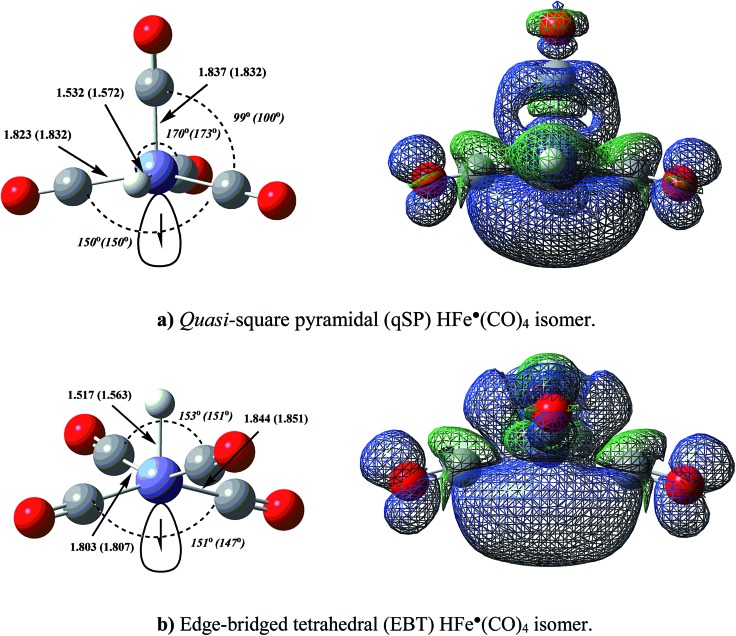
Equilibrium geometries of the HFe˙(CO)_4_ isomers (left), calculated at UB3LYP/BS-I and UCCSD/BS-III (data in parentheses) level of theory and isosurface plots of the corresponding singly occupied molecular orbitals (SOMOs) at ROB3LYP level (right). The SOMOs underscore that the unpaired electron has primarily metal-d_*z*_^2^ character (partially hybridized with ligand p_*z*_-orbitals) in both isomers, and that it is localized along the (a) *cis*- and (b) *trans*-directions to the corresponding Fe–H bonds, respectively. A Mulliken population analysis places 92% of the unpaired electron density on the Fe atom in the qSP and 82% in the EBT structure.

Carbon monoxide is a strong-field ligand in the spectrochemical series, and the metal in the HFe˙(CO)_4_ complex is thus in oxidation state +1 with a low-spin doublet (*S* = 1/2) electron configuration. Our analysis of the corresponding HFe˙(CO)_4_ PES is based on the following computational protocol: we performed a full geometry optimization using the BP86 functional^65^ (which is known as an economic path to accurate geometries in coordination compounds^56^) in conjunction with the LanL2TZ(f) effective core potential (ECP)^66^ for iron and the augmented double-*ζ* Dunning basis aug-cc-pVDZ^67^ for all other elements. We abbreviate this custom compound basis set as BS-I. To assess the quality of these baseline-level results, we reoptimized the geometries using the hybrid functional B3LYP and for selected cases the MP2 and CCSD level. The DFT results are in good agreement with those from CCSD, while MP2 shows larger discrepancies. MP2 is well-known to underestimate the metal carbonyl (M–CO) bond distances for the first-row transition metal complexes.^25,68,69^ For one of the isomers in this study, we obtained the following Fe–H bond distances, which confirm this issue: MP2: 1.35 Å; DFT: 1.52 Å; CCSD: 1.56 Å (*cf.* Fig. 3).

We also tested for basis set dependence by employing the all-electron augmented triple-*ζ* Pople basis 6-311+G(2d,p) (abbreviated BS-II), which is known to yield good geometries when combined with B3LYP.^70^ The differences were very minor, which demonstrates that BS-I is an adequate choice.

We performed frequency calculations at the same DFT levels to confirm the nature of each stationary point and to obtain thermodynamic corrections to the electronic energies (including zero-point energy corrections) and Gibbs free energies. Our study also employed internal reaction coordinate (IRC) calculations, for which the end point geometries were fully optimized to ensure connectivity between proper minima.

To obtain the best possible accuracy in the energetics, we performed high-level CCSD(T) single-point calculations on the stationary points obtained from B3LYP. The coupled cluster calculations employed the before-mentioned ECP for Fe and the standard double-*ζ* 6-31G(d,p)^71^ basis for the remaining elements. We abbreviate this basis set as BS-III. We also performed all-electron calculations with the BS-II basis set. To assess the degree of multireference character of a given system (which may limit the validity of CCSD(T) results), we performed T1-amplitude checks.

**Fig. 4:**
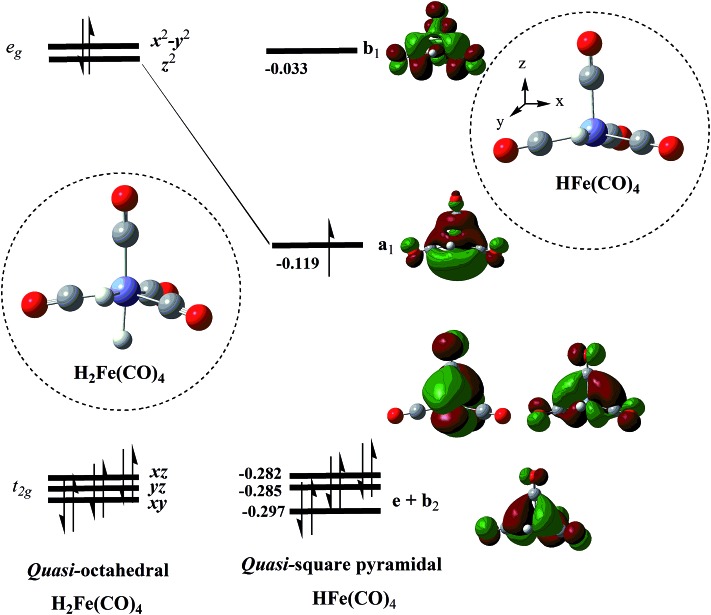
Orbital diagram demonstrating the formation of the low-spin d^7^-isolobal fragment-radical HFe(CO)_4_ *via* the removal of the axial H-ligand from the quasi-octahedral precursor H_2_Fe(CO)_4_. The hybrid orbital a_1_ in the HFe(CO)_4_ radical is pointed toward the vacant octahedral vortex. The orbital energies are in eV and were calculated at the ROB3LYP/BS-I level.

All calculations were carried out with the Gaussian 03 program package,^72^ utilizing the unrestricted, open-shell framework to account for the radical nature of the coordination compounds at hand. A restricted open-shell approach was used to create the radical single occupied molecular orbital (SOMO) plot in Fig. 3 and the orbital diagram in Fig. 4. We employed the fine grid option throughout, and an even finer grid (225 974 points) in selected cases to confirm the validity of small imaginary frequencies in Berry-type TSs. Excess spin densities were computed using the Mulliken population analysis,^73^ and we performed tests to assess the extent of basis-set dependence. The analysis results presented below are based on the largest basis set employed in this work, *i.e.*, BS-I.

The generally good agreement between the results obtained from different methods (see Table 1) – with the exception of MP2 – underscores their robustness and applicability, and thus supports the choices we made in designing our computational protocols. The distinct differences between the MP2 results and those of all other methods are readily explained based on the before-mentioned failure of MP2 to correctly describe the M–CO bonds in the systems at hand. Since the origin of the discrepancies is clear we will largely omit discussing the flawed MP2 results in Section 3. A comprehensive summary of all identified structures is provided as ESI (Table S1†).

**Table 1:** Potential energy surface parameters for polytopal rearrangements in the d^7^ radical complex HFe˙(CO)_4_ calculated at various levels of theory^*a*^

Stationary point^*b*^ (symmetry)	Calculation method^*c*^	Δ*E* ^*e*^ [kcal mol^–1^]	Δ*G* ^*e*^ [kcal mol^–1^]	Imaginary frequency [cm^–1^]
qSP (*C*_s_)	UBP86/BS-I	0.0	0.0	N/A
	UB3LYP/BS-I	0.0	0.0	N/A
	UB3LYP/BS-II	0.0	0.0	N/A
	UMP2/BS-I	0.0	0.0	N/A
	UCCSD(T)/BS-III//UB3LYP/BS-I^*d*^	0.0	0.0	N/A
	UCCSD(T)/BS-II//UB3LYP/BS-I^*d*^	0.0	0.0	N/A
EBT (*C*_2v_)	UBP86/BS-I	7.1	7.2	N/A
	UB3LYP/BS-I	8.7	8.8	N/A
	UB3LYP/BS-II	8.6	8.7	N/A
	UMP2/BS-I	10.5	10.6	N/A
	UCCSD(T)/BS-III//UB3LYP/BS-I^*d*^	10.2	10.3	N/A
	UCCSD(T)/BS-II//UB3LYP/BS-I^*d*^	10.7	10.8	N/A
TS_mBPR_ (*C*_4v_)	UBP86/BS-I	10.2 (3.1)	11.3 (4.1)	–60.4
	UB3LYP/BS-I	10.8 (2.1)	11.9 (3.1)	–50.7
	UB3LYP/BS-II	10.6 (2.0)	11.8 (3.1)	–48.3
	UMP2/BS-I	34.6 (24.1)	35.2 (24.6)	–182.8
	UCCSD(T)/BS-III//UB3LYP/BS-I^*d*^	17.2 (7.0)	18.2 (8.0)	—
	UCCSD(T)/BS-II//UB3LYP/BS-I^*d*^	13.8 (3.1)	14.9 (4.1)	—
TS_OSH_ (*C*_2v_)	UBP86/BS-I	2.9	3.0	–490.9
	UB3LYP/BS-I	2.7	2.8	–444.6
	UB3LYP/BS-II	2.7	2.9	–443.8
	UMP2/BS-I	8.5	8.6	–905.1
	UCCSD(T)/BS-III//UB3LYP/BS-I^*d*^	5.3	5.4	—
	UCCSD(T)/BS-II//UB3LYP/BS-I^*d*^	3.7	3.8	—
TS_OSL_ (*C*_2v_)	UBP86/BS-I	4.2	4.4	–337.4
	UB3LYP/BS-I	4.1	4.3	–335.0
	UB3LYP/BS-II	3.9	4.2	–298.6
	UMP2/BS-I	6.3	6.9	–123.1
	UCCSD(T)/BS-III//UB3LYP/BS-I^*d*^	5.2	5.5	—
	UCCSD(T)/BS-II//UB3LYP/BS-I^*d*^	6.1	6.4	—
TS_BFI_ (*C*_s_)	UBP86/BS-I	8.3	8.5	–420.1
	UB3LYP/BS-I	9.5	9.7	–422.4
	UB3LYP/BS-II	9.3	9.5	–402.0
	UMP2/BS-I	11.3	11.2	–600.2
	UCCSD(T)/BS-III//UB3LYP/BS-I^*d*^	10.2	10.3	—
	UCCSD(T)/BS-II//UB3LYP/BS-I^*d*^	11.1	11.3	—

^*a*^Relative electronic energies (Δ*E*) include zero-point energy corrections; Gibbs free energies (Δ*G*) are for 298.15 K.

^*b*^qSP: quasi-square pyramidal isomer; EBT: edge-bridged tetrahedral isomer; TS_mBPR_: modified Berry pseudorotation transition state; TS_OSH_: octahedral hydrogen shift (switch) transition state; TS_OSL_: octahedral ligand shift (switch) transition state; TS_BFI_: butterfly isomerization transition state.

^*c*^BS-I basis set represents LanL2TZ(f) for Fe and aug-cc-pVDZ for all other elements; BS-II: 6-311+G(2d,p); BS-III: LanL2TZ(f) for Fe, 6-31G(d,p) for all other elements.

^*d*^CCSD(T) energies are corrected with zero-point vibration energies (Δ*E*), and thermal coefficients (Δ*G*) at UB3LYP/BS-I level.

^*e*^The values in brackets are relative to the higher-energy EBT isomer, to which these TSs refer (see Fig. 5 and 9).

## 3. Results and discussion

### 3.1 Isomers of HFe˙(CO)_4_

Based on the EPR data of Krusic and co-workers as well as an extended Hückel theory analysis, HFe˙(CO)_4_ has previously been assigned an SP equilibrium geometry of *C*_4v_ symmetry with the hydrogen located in the apical position.^47–49^ No evidence has been reported to support a TBP structure with an axial H-ligand. Our first-principles characterization of HFe˙(CO)_4_ and its PES are summarised in Table 1. None of the presented results (at any level of theory) support the postulated SP equilibrium structure, nor do they support the other ideal penta-coordination polyhedron, *i.e.*, a TBP isomer. Rather than being a minimum on the PES, we have identified the SP structure to be a low-lying saddle point. The true minimum is a quasi-SP (qSP) structure of *C*_s_ symmetry with a basal hydrogen (see Fig. 3a), which is in accord with the geometries reported for a number of HM(CO)_4_ complexes.^23^ In addition to the qSP equilibrium structure, an edge-bridged tetrahedron (EBT) of *C*_2v_ symmetry (see Fig. 3b) emerged as another relevant isomer.

EBT structures are well-known and constitute a separate class of penta-coordinate complexes.^19,27,31^

The distorted, low-symmetry qSP equilibrium geometry is about 10 kcal mol^–1^ more stable than the EBT isomer. Our analysis of the spin density distribution (Fig. 3) shows some differences in the directionality and localization of the metal-centered radical electron between the qSP and EBT isomers: both are more pronounced for the former, which may be attributed to a less hindered Fe-center and which may indicate a relatively larger reactivity.^15^ HFe˙(CO)_4_ can be expected to exhibit a high propensity for dimerization,^48^ which has been confirmed experimentally by the formation of the bimetallic H_2_Fe_2_(CO)_8_ dimer. Its geometry represents two qSP structures linked to each other *via* radical centers,^47–49^ which is consistent with the above consideration.

There are several ways in which we can rationalize the computed qSP and EBT isomer structures of HFe˙(CO)_4_. One way is to employ Hoffmann's isolobal concept,^4^ according to which HFe˙(CO)_4_ can be represented as an isolobal analogue of the d^7^-fragment of an ML_6_ complex, such as Mn(CO)_5_ and Co(CN)_5_^3–^. (Hoffmann actually considered the *C*_4v_ M(CO)_5_ fragment formation *via* the removal of an axial ligand from an octahedral precursor when developing this concept.^20,21^)

If we consider an idealized *O*_h_ H_2_Fe(CO)_4_ precursor and remove an axial H, then this strong perturbation is expected to shift one of the metal–ligand σ-antibonding orbitals of the degenerate *e*_g_ set to lower energies, thus creating a low-lying acceptor orbital in the emerging qSP radical. The DFT results shown in Fig. 4 illustrate this notion. It is worth noting that d^6^ HMn(CO)_4_ also adopts a qSP structure, however, in this case the H occupies a basal position. In contrast to HFe˙(CO)_4_, HMn(CO)_4_ also features a less stable SP isomer with the H in apical position.^23^

As an alternative precursor for HFe˙(CO)_4_, we can consider the diamagnetic [HFe(CO)_4_]^–^ with d^8^ electron configuration. The anion exhibits a TBP-type structure,^74,75^ for which we can assign an idealized *D*_3h_ symmetry. If we remove one electron to furnish HFe˙(CO)_4_, this would leave one unpaired electron in a degenerate e′-orbital.^20,21^ The resulting Jahn–Teller distortion^19,76,77^ would lift the degeneracy of this set of orbitals and lead to the formation of the qSP and EBT structures observed for HFe˙(CO)_4_. This distortion should occur along a reverse-BPR pathway, *i.e.*, upon upward bending of the two axial ligands **3** and **4** in the direction of the pivotal ligand **5** as shown in Fig. 1. Ward *et al.* have reported such an EBT distortion in d^0^ MD_2_L_3_-type compounds containing two equatorial π-donor ligands D.^27^ Another pathway to distort the ideal *D*_3h_ symmetry and lift the degeneracy of the e′-orbitals may proceed through the formation of distorted-TBP (dTBP) structures, which exhibit an acute angle *α* in the equatorial plane.^28^ Our calculations identified such a dTBP-type geometry, albeit for the transition structures (see Fig. 6 and 7).

Despite all this evidence, Jahn–Teller-type distortions alone do not suffice to explain why HFe˙(CO)_4_ adopts an EBT structure for the second isomer. This becomes evident when we consider the electronically analogous complexes of the heavier homologues Ru and Os, which adopt SP rather than EBT geometries (see Table 2). We will revisit and discuss this issue in more detail in Section 3.6.

**Table 2:** Comparative data on polytopal rearrangements in d^7^ HM(CO)_4_ systems^*a*^

Compound (configuration)	Stationary point	Point group symmetry	Δ*E* ^*d*^ [kcal mol^–1^]	Imaginary frequency [cm^–1^]
[HCo(CO)_4_]˙^+^ (3d^7^)	qSP	*C*_s_	0.0	N/A
	EBT	*C*_2v_	10.7	N/A
	TS_mBPR_	*C*_4v_	20.2 (9.5)	–250.2
	TS_OSH_	*C*_2v_	7.2	–728.4
	TS_OSL_	*C*_2v_	4.3	–297.5
	TS_BFI_	*C*_s_	10.6^*e*^	–183.2
HFe˙(CO)_4_ (3d^7^)	qSP	*C*_s_	0.0	N/A
	EBT	*C*_2v_	9.7	N/A
	TS_mBPR_	*C*_4v_	12.8 (3.1)	–65.8
	TS_OSH_	*C*_2v_	3.2	–465.5
	TS_OSL_	*C*_2v_	3.9	–333.3
	TS_BFI_	*C*_s_	10.1	–405.8
[HMn(CO)_4_]˙^–^ (3d^7^)	qSP	*C*_s_	0.0	N/A
	EBT	*C*_2v_	8.5	N/A
	TS_mBPR_	*C*_4v_	13.8 (5.3)	–64.3
	TS_OSH_ ^*c*^	*C*_2v_	0.8	–280.7
	TS_OSL_	*C*_2v_	3.5	–292.8
	TS_BFI_	*C*_s_	8.8	–416.5
HRu˙(CO)_4_ (4d^7^)	qSP	*C*_s_	0.8	N/A
	SP (*θ* = 91.2°)^*b*^	*C*_4v_	0.0	N/A
	TS_OSH_	*C*_2v_	7.8	–623.0
	TS_OSL_	*C*_2v_	5.8	–227.0
	TS_BFI_	*C*_s_	7.5	–331.8
HOs˙(CO)_4_ (5d^7^)	qSP	*C*_s_	0.3	N/A
	SP (*θ* = 91.8°)^*b*^	*C*_4v_	0.0	N/A
	TS_OSH_	*C*_2v_	5.9	–555.4
	TS_OSL_	*C*_2v_	6.3	–210.4
	TS_BFI_	*C*_s_	8.1	–355.4

^*a*^Zero-point energy-corrected relative electronic energies (Δ*E*) and frequencies at UB3LYP/BS-III level of theory.

^*b*^Apical H and *θ* droop angle.

^*c*^Similar to a mechanism suggested by Church *et al.* ^23^

^*d*^The values in brackets are relative to the higher-energy EBT isomer, to which these TSs refer.

^*e*^The small decrease of the TS_BFI_ energy compared to the EBT minimum stems from the imperfect harmonic oscillator approximation used to calculate the zero-point energy correction. The electronic energy of TS_BFI_ is higher than EBT (albeit by a small amount of 0.05 kcal mol^–1^).

A number of higher symmetry structures, including the aforementioned SP, emerge as saddle points on the PES. They connect the (stereo-)isomers and thus represent TSs of the corresponding polytopal rearrangements. We have identified two main mechanisms for fluxional rearrangement, *i.e.*, a modified BPR (mBPR) and – due to its conceptual similarity to Muetterties' TJ – an octahedral switch (OS). For the interconversion between the non-equivalent qSP and EBT structures we discovered a butterfly isomerization (BFI) mechanism. It is worth noting that – consistent with our discussion in Section 1 and ref. 24, 41 and 42 – no TS for a TSR mechanism could be found on the HFe˙(CO)_4_ PES.

### 3.2 Modified Berry pseudorotation

**Fig. 5:**
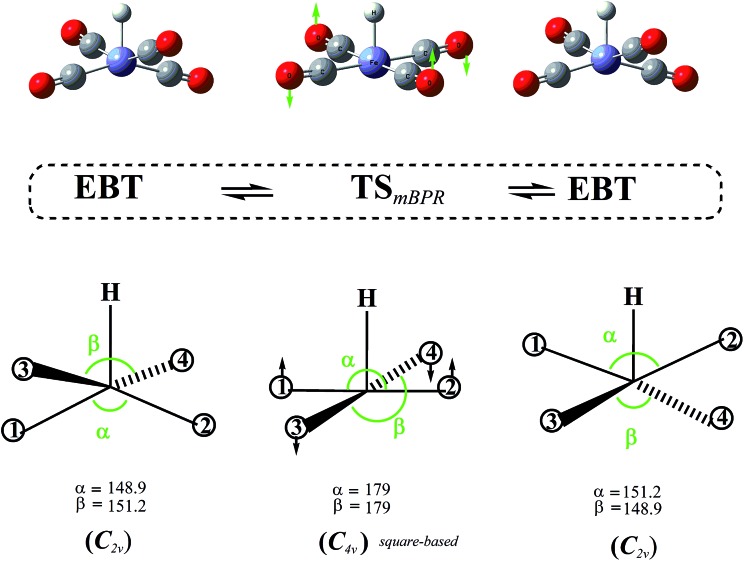
Modified Berry pseudorotation (mBPR) mechanism interconverting two edge-bridged tetrahedral (EBT) stereoisomers of *C*_2v_ symmetry *via* a transition state (TS) of *C*_4v_ symmetry.

As indicated in the previous section, our calculations show that the symmetric SP structure is not a minimum but a saddle point on the HFe˙(CO)_4_ PES. Closer inspection reveals that it interconverts two EBT stereoisomers, and the corresponding trajectory points to a BPR-type pathway (*i.e.*, two equatorial ligands are simultaneously moving up, another two down, and *vice versa;* see Fig. 5).

However, unlike for the classical BPR mechanism (Fig. 1), no high-symmetry TBP structures could be identified to represent the end-points of the inversion. An IRC analysis of the TS yielded the two EBT stereoisomers as the true end-point structures instead. The DFT barrier heights (Δ*E*, which include zero-point corrections) for this mBPR mechanism range from 2.0 to 3.1 kcal mol^–1^ (see Table 1). The corresponding Gibbs free energies (Δ*G*) are somewhat higher (by about 1 kcal mol^–1^) due to the entropy decrease in the high-symmetry TS_mBPR_. (This effect is not seen for the other mechanisms described below, for which the values for Δ*E* and Δ*G* are nearly identical.) The computed barriers are close to the experimental activation energy of 1.6 ± 0.3 kcal mol^–1^ for the classical BPR in Fe(CO)_5_,^36^ which suggests that our results are reasonable. Our single-point all electron CCSD(T) calculations predict a similar value for Δ*E* (3.1 kcal mol^–1^), whereas the ECP-based calculations yielded a somewhat higher barrier of 7.0 kcal mol^–1^.

### 3.3 Octahedral switch

**Fig. 6:**
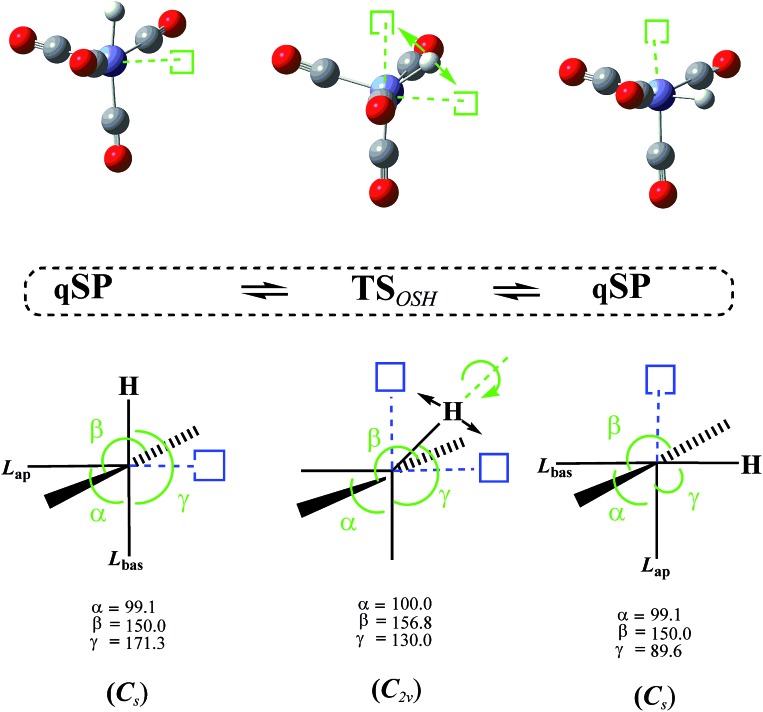
Octahedral switch mechanism for hydrogen (OSH) interconverting two quasi-square pyramidal (qSP) stereoisomers of *C*_s_ symmetry *via* an edge-bridged tetragonal (EBT) transition state (TS) of *C*_2v_ symmetry. The basal H-ligand of the qSP structure (idealized as a half-octahedron in the schematic for ease of representation) migrates to the vacant octahedral position, interchanging the apical and basal CO-ligands (denoted as L_ap_ and L_bas_) through an inversion of the whole molecule. Vacant octahedral sites (open squares) and apical-basal interchanging ligands are highlighted. Key angles of the distorted structures are provided.

**Fig. 7:**
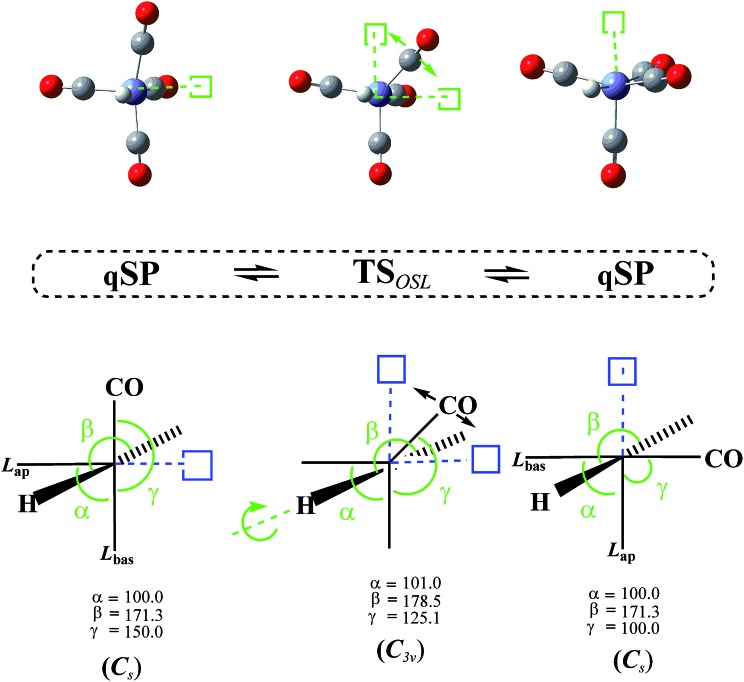
Octahedral switch mechanism for the CO-ligand (OSL) interconverting two quasi-square pyramidal (qSP) stereoisomers of *C*_s_ symmetry *via* a distorted trigonal bipyramidal (dTBP) transition state (TS) of *C*_2v_ symmetry. A basal CO-ligand of the qSP structure (idealized as a half-octahedron in the schematic for ease of representation) migrates to the vacant octahedral position, interchanging the apical and basal CO-ligands (denoted as L_ap_ and L_bas_) through an inversion of the whole molecule. Vacant octahedral sites (open squares) and apical-basal interchanging ligands are highlighted. Key angles of the distorted structures are provided.

In addition to the TS_mBPR_, we have located two saddle points on the HFe˙(CO)_4_ PES that each connect a pair of chemically identical qSP stereoisomers. Both pathways of this octahedral switch mechanism describe a single ligand migration to a vacant position of an “octahedron” *via* a TS of *C*_2v_ symmetry. The initial qSP structure is considered the idealized, non-vacant part of this octahedron. We distinguish the cases where (i) H migrates from a basal position of the qSP to the vacant position of the octahedron *via* an EBT TS (this case is denoted as OSH; see Fig. 6), and (ii) a bulkier CO migrates to the same vacant octahedral position *via* a TS of dTBP geometry (this case is denoted as OSL; see Fig. 7). Unlike the TJ mechanism described in Fig. 2, in which the ligand migration occurs between faces of the tetrahedron, the OS mechanism involves ligand migration between (open) vertices of the “octahedron”, resulting in the apical-basal ligand exchange (switch).

It is worth noting that the OSH mechanism has some resemblance to the pivoting mechanism suggested by Albright *et al.* for penta-coordinate d^6^-complexes,^19^ except that the latter includes a pivoting of two ligands, whereas in the former, only one ligand is moving. In the OSL, two other equatorial ligands of the dTBP TS are also moving concurrently in opposite directions to maintain zero net angular momentum in a manner to support the OS and the respective stereoisomer formation. This phenomenon resembles that described by Berke, Gusev, and co-workers for Ir(H)_2_X(P^*t*^Bu_2_Ph) penta-coordinate systems, where X is a halogen.^11,29,78^ However, in contrast to the OSL mechanism, the X-ligand in ref. 29 does not change its position. For the OSH mechanism we found even closer parallels to early work in the field. While Burdett *et al.* proposed the inverse Berry twist (SP → [TBP]^‡^ → SP′) involving singlet excited states to explain the photochemical behavior of d^6^ M(CO)_5_ complexes,^14^ they also postulated an alternative mechanism involving a *C*_2v_ TS. However, based on extended Hückel theory calculations and the resulting d-orbital stabilization energies, it was concluded at the time that repulsive interactions would make such a TS unfavorable compared to the TBP TS for d^7^ and d^8^ complexes, and that it could only be relevant for d^6^ low-spin complexes. The OS mechanism is also somewhat reminiscent of the fluxionality of “clusters” that feature a vacant site on a polyhedral surface^79–83^ following the simple bond-stretch isomerism as postulated by Stohrer and Hoffmann.^84^ Such an isomerization occurs, *e.g.*, between two identical SP intermediates of the (C_5_H_5_)^+^ cation *via* a *C*_2v_ TS, which was predicted at the MINDO/3,^85^ MP2,^86^ and DFT^82,83^ levels. Saillard and co-workers have calculated this “migration” for the isoelectronic P_5_^+^ and Sb_5_^+^ as well as B-substituted clusters.^82,83^ Even though the bond-breaking processes in these “hollow” clusters require only relatively low activation energies (30 kcal mol^–1^ at MP2/DZP^86^), they are still significantly higher than those for OS rearrangements in metallocomplexes.

The DFT-level Δ*E* for the OSH mechanism range from 2.7 to 2.9 kcal mol^–1^ and for the OSL from 3.9 to 4.2 kcal mol^–1^ (the corresponding Δ*G* are only very slightly higher, *i.e.*, by about 0.2 kcal mol^–1^; see Table 1), *i.e.*, the OS barriers for the light H-atom is lower by about 1 kcal mol^–1^ than the one for the heavier CO.

The MP2 results favor the OSL mechanism with a barrier of 6.3 kcal mol^–1^ over the OSH mechanism with 8.5 kcal mol^–1^. The ECP-based CCSD(T)/BS-III results are essentially degenerate with OSL narrowly winning out (5.2 *vs.* 5.3 kcal mol^–1^), whereas our highest level results from the all-electron CCSD(T)/BS-II calculations confirm the DFT predictions favoring the H-shift (3.7 *vs.* 6.1 kcal mol^–1^). Considering the small energetic differences and the method inherent margins of error, we cannot make a conclusive statement concerning the order of these two rearrangement pathways.

### 3.4 Butterfly isomerization

**Fig. 8:**
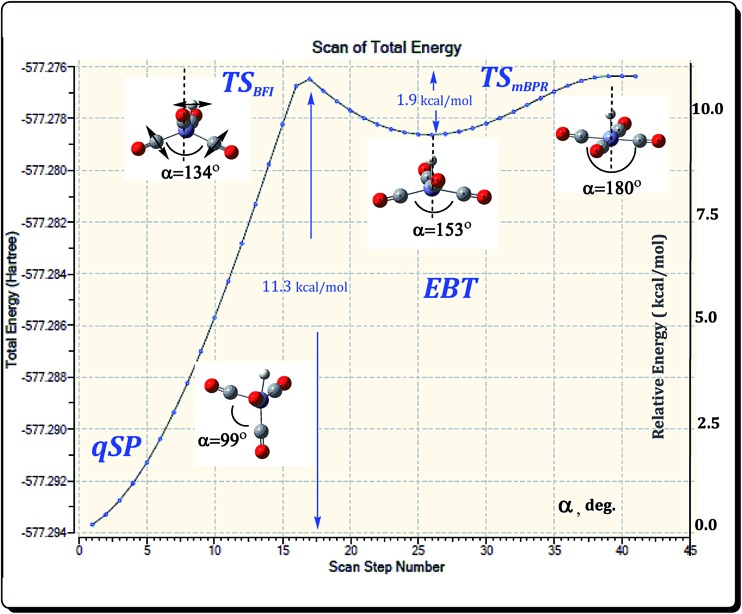
Energetics at UB3LYP/BS-I level for the acute angle *α* scan starting from the quasi-square pyramidal (qSP) HFe˙(CO)_4_ isomer. Relative to qSP energies in kcal mol^–1^ (Δ*E*), are shown for clarity. The scan offers a simple approximation of consecutive rearrangement steps.

The mBPR and OS mechanisms described so far facilitate fluxional rearrangements that connect chemically indistinguishable species, *i.e.*, qSP to qSP′ and EBT to EBT′, respectively. There also has to be a mechanism that interconverts the qSP isomer into the EBT and *vice versa*. This mechanism should intuitively include the migration of H or CO-ligands, as well as deformation of the acute angle *α*. An examination of the two distinct HFe˙(CO)_4_ isomers (see Fig. 3) suggests that the isomerization could simply proceed through a small shift of the H position. However, a direct H-migration scan (see Fig. S1, ESI†) reveals that such a pathway does not end up at the EBT isomer, but rather the TS_OSH_, which has a similar EBT geometry. The only difference is the acute angle *α*. On the other hand, a variation of *α* provides a simple picture of consecutive transformations, and the results of such an *α*-scan are presented in Fig. 8.

The first maximum of the energy profile is an approximate representation of the TS for the qSP to EBT isomerization. It features a distorted EBT (dEBT) geometry of *C*_s_ symmetry (with asymmetrical wing angles being 125° and 101°, respectively) and a barrier height of about 11 kcal mol^–1^. As expected, the *α* angle has an intermediate value of 134° between that of the two isomers (99° and 153° for qSP and EBT, respectively). The shallow minimum (well-depth of about 1–2 kcal mol^–1^) and small reverse barrier indicate that the EBT is kinetically unstable. Since the EBT is also the higher-energy (*i.e.*, thermodynamically unstable) isomer, it is expected that it will have a transient character. A further increase of *α* leads to a regular SP structure, which corresponds to the TS_mBPR_ that interconverts EBT stereoisomers as discussed in Section 3.2. The actual TS given in Table 1 was determined following the *α*-scan by full gradient norm optimization. Since this TS combines the before-mentioned H-migration with a butterfly-type motion of the CO-ligands, we call this the butterfly isomerization (BFI) mechanism. The Δ*E* for the BFI mechanism range from 8.3 to 9.5 kcal mol^–1^ at the DFT level (Δ*G* are 0.2 kcal mol^–1^ higher in each case), and between 10.2 and 11.1 kcal mol^–1^ at the MP2 and CCSD(T) levels (see Table 1). The BFI is thus energetically more demanding than the fluxional rearrangements as they involve more significant molecular deformations.

### 3.5 The HFe˙(CO)_4_ PES

**Fig. 9:**
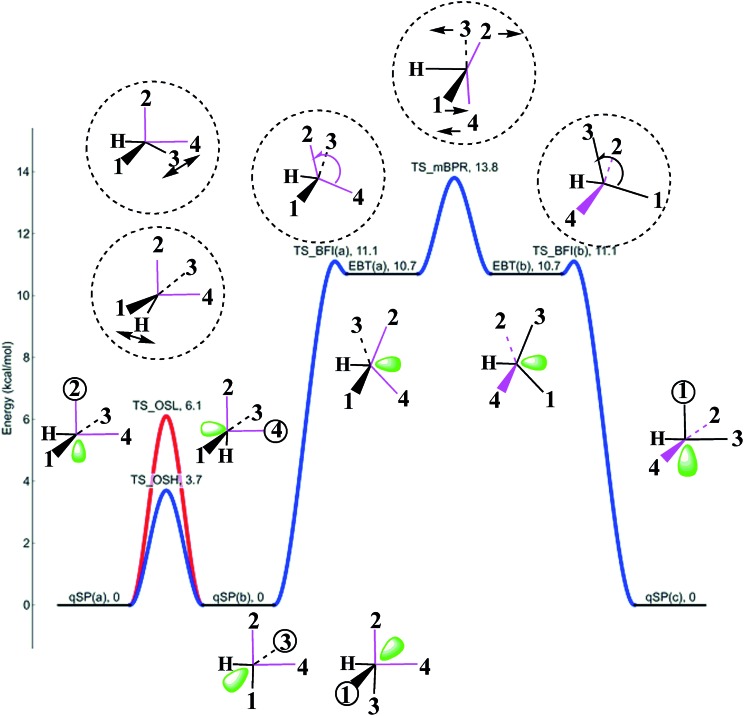
Potential energy plot for the polytopal rearrangements in HFe˙(CO)_4_ calculated at the UCCSD(T)/BS-II//UB3LYP/BS-I level of theory. qSP(a), qSP(b), qSP(c) represent chemically indistinguishable quasi-square pyramidal stereoisomers, EBT(a), EBT(b) edge-bridged tetrahedral ones. TS_OSH, TS_OSL, TS_BFI(a), TS_BFI(b), TS_mBPR, denote transition states (TS) for the two octahedral switch processes (OSH, OSL), two equivalent butterfly isomerizations (BFI), and modified Berry pseudorotation (mBPR). TS structures and the apical ligand numbers are encircled and the relative orientation of ligands **2** and **4** highlighted in pink to emphasize the characteristic structural changes. See Fig. 6–8, for details on each individual process. The bottom two structures were obtained from two similar TS_OSL_ structures (one omitted for clarity) that involve ligands **3** and **1**, respectively.

Fig. 9 shows a schematic representation of the HFe˙(CO)_4_ PES with all the stationary points introduced in the previous subsections. The qSP minimum is the stable isomer of HFe˙(CO)_4_, but it can undergo fluxional stereoisomerization, either *via* one of the OS pathways or a BFI → mBPR → BFI′ sequence with metastable EBT stereoisomers as intermediates. Since the consecutive rearrangement results in a relatively high overall barrier (13.8 kcal mol^–1^ at all electron CCSD(T) level), the OS will be the dominant process at low temperatures. It is worth stressing that the different mechanisms lead to the rearrangement of different ligands. In fact, the polytopal rearrangements can be traced through the changes in the apical positions of the ligands (the corresponding ligand number is encircled for clarity in Fig. 9) as well as the relative orientation of ligands **2** and **4** (highlighted in pink).

The OS stereoisomerization is shown on the left side of the PES diagram. It features a swap in the apical and basal ligand positions, while the corresponding ligands **2** and **4** remain *cis*-oriented to each other and the angular orientation of the H-atom with respect to **2** and **4** does not change. In the processes on the right side, the H-atom is the pivoting ligand, and **2** and **4** rearrange from *cis*- to *trans*-orientation. The latter can only be achieved through the energetically less favorable BFI → mBPR → BFI′ sequence, which is only accessible at more elevated temperatures. Only a traversal of the full sequence (facilitated by the deformation of *α*) inverts the **1–3** *trans*- and **1–4** *cis*-orientation and replaces the **2**-H *trans*- with the **3**-H *trans*-orientation. It would be an interesting (albeit challenging) task for experimentalists to discriminate these steps to prove the theoretical conclusions drawn from this study.

### 3.6 Comparison with other d^7^ HM˙(CO)_4_ systems

The isomers (*i.e.*, qSP and EBT) and mechanisms (*i.e.*, mBPR, OSH, OSL, and BFI) introduced in Sections 3.1–3.4 for the prototype system HFe˙(CO)_4_ may offer a more general picture of polytopal arrangements in five-coordinate systems, at least for d^7^-complexes. We have thus expanded our DFT study of the HFe˙(CO)_4_ radical to an initial set of related coordination compounds. Systems that are electronically homologous to HFe˙(CO)_4_ but that feature different central metal atoms are an obvious first choice for a comparative study. In the following, we will provide such a comparison and discuss the transferability of our findings. The results of this work are summarized in Table 2.

All 3d^7^-systems studied here, *i.e.*, [HCo(CO)_4_]˙^+^, HFe˙(CO)_4_, and [HMn(CO)_4_]˙^–^, feature qSP isomers as the global minima on their respective PESs and they represent the end-point structures for OS-rearrangements (see Fig. 9). Each example also features a metastable EBT isomer between 8.5 and 10.7 kcal mol^–1^ above the qSP structure. EBT stereoisomers interconvert *via* the proposed mBPR mechanism in all cases. Finally, we also find the BFI mechanism as the mode of isomerization between the qSP and EBT isomers in each of our 3d^7^ examples. Our discussion in Sections 3.1–3.5 is thus fully applicable for the other 3d^7^-systems considered here as well. While the three 3d^7^-compounds have identical electron configurations, they differ in their nuclear charges. The barrier heights for the polytopal rearrangements correlate strongly with the nuclear charge, *i.e.*, they are highest for the Co and lowest for the Mn case. This is consistent with our expectation for electrostatically induced rigidity in these complexes (*i.e.*, the electrons in the bonding orbitals are subject to a stronger electrostatic attraction from the nucleus – exacerbated by the decreasing atomic radii from Mn to Fe to Co – which makes the ligands less mobile and susceptible to rearrangements).

The two remaining d^7^ radicals in the iron group (*i.e.*, HRu˙(CO)_4_ and HOs˙(CO)_4_) also feature qSP structures, but instead of the additional EBT isomers, they adopt symmetric SP structures with H in the apical position. EBT isomers and the corresponding mBPR stereoisomerization pathways found in the 3d^7^-systems could not be identified in the 4d^7^ and 5d^7^ cases. The SP structures are marked by relatively large apical-M-basal angles of 91.2° and 91.8° for Ru and Os, respectively. The qSP structures are found to be slightly higher in energy (*i.e.*, less stable) than the SP isomers by 0.7 and 1.7 kcal mol^–1^, respectively, *i.e.*, for the Ru and Os radical complexes the qSP structures are not the global minima on the PES. (However, considering the small energetic differences and inherent margins of error of DFT, this is not a conclusive finding.) The isomerization from qSP to SP proceeds through a BFI-type mechanism.

The preference of the SP over the EBT structure may be traced back to the differences in the covalent radii of the metal centers (Ru: 1.37 Å; Os: 1.38 Å; Fe: 1.24 Å (ref. 87)). Classical interligand interaction theory^10,18^ based on the partitioning of the interligand interactions into 6-exp (one-variable Kitaigorodsky-type) and Coulomb potentials makes it possible to distinguish the contributions of size and polarity of ligand spheres, respectively, to the stabilization of a polyhedral structure. Qualitatively, the increase in the covalent radius of the central metal (assuming fixed valence-shell configuration) results in longer M–L coordination bonds (*i.e.*, a larger coordination sphere) and consequently in an increased interligand spacing. Such an increased spacing (which would be expected for the Ru and Os complexes relative to the Fe prototype) will alleviate steric repulsion between the ligands and will thus make coordination polyhedra with smaller baseline ligand separation (such as SP in comparison to EBT) more competitive.^10^

We point out that all isomer energy differences and all barriers heights of the d^7^ systems shown in Table 2 are smaller than 11 kcal mol^–1^ and thus energetically very close. The presence of such shallow PESs and closely related stationary points underscores the challenges of studying the nature, mechanistic intricacies, and impact of polytopal rearrangements.

## 4. Conclusions

Using an array of DFT and *ab initio* approaches, we have demonstrated that the penta-coordinate d^7^ complex HFe˙(CO)_4_ has two thermally accessible isomers – a qSP equilibrium structure and a metastable EBT – and that the ideal high-symmetry SP geometry that was advocated in earlier work is in fact not a stable structure but a TS. We can rationalize the lower symmetry qSP and EBT structures as being derived from isolobal, higher symmetry precursors that become subject to Jahn–Teller-type distortions upon transition to the d^7^ radical, which would otherwise feature a degenerate orbital occupied by a single electron.

Both isomers are subject to fluxional rearrangements (*i.e.*, stereoisomerization) as well as interconversion (*i.e.*, isomerization) during which they traverse different polyhedral forms. We have analysed these intramolecular polytopal rearrangement processes for the HFe˙(CO)_4_ prototype system and could identify three new mechanisms that facilitate them: mBPR, OS, and BFI. We could also show that the host of other mechanisms that have been proposed for this class of compounds in the past are ultimately not applicable. Our study thus fills a gap in the mechanistic understanding of rearrangement processes for systems involving distorted equilibrium structures. We have characterized and rationalized each mechanism in detail and shown that they are valid for other 3d^7^-systems as well. The d^7^-complexes of the higher rows exhibit a somewhat different situation with an SP equilibrium geometry and a metastable qSP isomer. The discrepancy may be attributed to the weaker interligand interactions found in systems with larger coordination spheres.

The barrier heights for all mechanisms are on the order of 10 kcal mol^–1^ or smaller, and the barrier differences are within a few kcal mol^–1^. We note that some of these differences are basically within the margin of error of the applied computational methods, so that we cannot claim a definite order in these cases. The shallow nature of the PESs of the systems at hand indicates that experimental studies on the prevalent mechanisms will be challenging.

In upcoming publications, we will investigate the impact of the nature of the ligand (in particular its electronic structure and propensity for different types of coordinative bonds) on these newly proposed polytopal rearrangement mechanisms, and we will also expand the scope of our investigation to penta-coordinated complexes with d^6^ and d^8^ electron configuration.
