# Supplementary material for: Revisiting the polytopal rearrangements in penta-coordinate d7-metallocomplexes: modified Berry pseudorotation, octahedral switch, and butterfly isomerization
Source: Chem Sci. 2017 Jun 2;8(8):5512–25. doi: 10.1039/c7sc00703e (PMC5618771; doi:10.1039/c7sc00703e)
Supplement: Supplementary file 1 [file SC-008-C7SC00703E-s001.pdf]

Electronic Supplementary Material (ESI) for Chemical Science.
This journal is © The Royal Society of Chemistry 2017

SUPPORTING INFORMATION

Revisiting the Polytopal Rearrangements in Penta-Coordinate d7-Metallocomplexes: Modified Berry Pseudorotation, Octahedral Switch, and Butterfly Isomerization

Rubik Asatryan, Johannes Hachmann, and Eli Ruckenstein

Department of Chemical and Biological Engineering, State University of New York

Buffalo, NY, 14260

*****

Figure S1. Direct relaxed scan of the C-Fe_H angle from the qSP equilibrium state leading to TS_{OSH}.

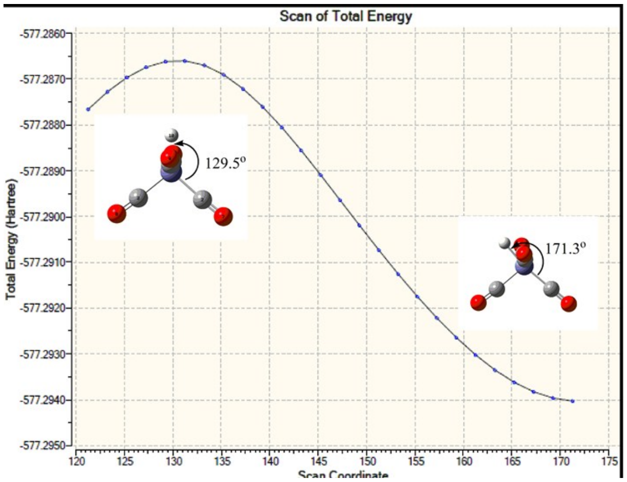

(see text, Sec. 3.4, Butterfly Isomerization.).

*****

Full Citation for Ref. [72]

Gaussian 03, Revision D.01, M. J. Frisch, G. W. Trucks, H. B. Schlegel, G. E. Scuseria, M. A. Robb, J. R. Cheeseman, J. A. Montgomery, Jr., T. Vreven, K. N. Kudin, J. C. Burant, J. M. Millam, S. S. Iyengar, J. Tomasi, V. Barone, B. Mennucci, M. Cossi, G. Scalmani, N. Rega, G. A. Petersson, H. Nakatsuji, M. Hada, M. Ehara, K. Toyota, R. Fukuda, J. Hasegawa, M. Ishida, T. Nakajima, Y. Honda, O. Kitao, H. Nakai, M. Klene, X. Li, J. E. Knox, H. P. Hratchian, J. B. Cross, V. Bakken, C. Adamo, J. Jaramillo, R. Gomperts, R. E. Stratmann, O. Yazyev, A. J. Austin, R. Cammi, C. Pomelli, J. W. Ochterski, P. Y. Ayala, K. Morokuma, G. A. Voth, P. Salvador, J. J. Dannenberg, V. G. Zakrzewski, S. Dapprich, A. D. Daniels, M. C. Strain, O. Farkas, D. K. Malick, A. D. Rabuck, K. Raghavachari, J. B. Foresman, J. V. Ortiz, Q. Cui, A. G. Baboul, S. Clifford, J. Cioslowski, B. B. Stefanov, G. Liu, A. Liashenko, P. Piskorz, I. Komaromi, R. L. Martin, D. J. Fox, T. Keith, M. A. Al-Laham, C. Y. Peng, A. Nanayakkara, M. Challacombe, P. M. W. Gill, B. Johnson, W. Chen, M. W. Wong, C. Gonzalez, and J. A. Pople, Gaussian, Inc., Wallingford CT, 2004.

*****

Table S1. Geometries and Frequencies of $HFe(CO)_4$ Radical Calculated at Different Levels Presented in Table 1.

UBP86/LanL2TZ(f) ECP[Fe]/ aug-cc-*p*VDZ [C,O,H] *(BS-I)*

Quasi-SP

Charge = 0 Multiplicity = 2

Cartesian Coordinates

26	0.000008	-0.025568	-0.305942
6	-0.000030	1.558936	0.579702
6	0.000013	-1.117223	1.121329
6	1.742169	-0.151678	-0.759653
6	-1.742146	-0.151723	-0.759671
8	2.847913	-0.303200	-1.080997
8	-0.000080	2.572699	1.147131
8	0.000015	-1.836956	2.034184
8	-2.847880	-0.303284	-1.081033
1	0.000028	-1.199183	-1.290039

Frequencies, cm^{-1}

68.4396	79.6159	88.4631
92.9108	98.7021	308.3970
350.7729	356.0121	425.5139
445.8352	474.4454	478.6935
491.4346	518.5554	527.5544

558.6573 594.7186 677.8909
705.6592 1886.1504 1981.8608
1984.0827 1993.8076 2057.0068

EBT

Charge = 0 Multiplicity = 2

Cartesian Coordinates

26	0.000003	-0.000001	-0.030909
6	-0.008716	-1.727252	0.426397
6	0.008707	1.727274	0.426310
6	-1.758043	0.008984	-0.503837
6	1.758059	-0.009012	-0.503795
8	-2.879878	0.015040	-0.807403
8	-0.014242	-2.820526	0.823519
8	0.014226	2.820566	0.823380
8	2.879883	-0.015080	-0.807405
1	-0.000027	0.000043	1.476448

Frequencies, cm^{-1}

54.7063 87.4244 89.9554
91.9589 105.0148 329.7259
371.8623 387.6874 415.7155
458.3961 483.1697 485.2348
490.2882 523.7436 551.6471
583.1640 616.9070 638.9542
681.2923 1915.0712 1965.0095
1980.2404 1983.8952 2058.4764

TS$_{mBPR}$

Charge = 0 Multiplicity = 2

Cartesian Coordinates

26	0.000005	-0.000013	-0.032881
6	-0.575347	-1.708435	-0.039583
6	0.575327	1.708420	-0.039602
6	-1.708417	0.575341	-0.039566
6	1.708438	-0.575334	-0.039626
8	-2.808722	0.945960	0.010693
8	-0.945965	-2.808741	0.010665
8	0.945905	2.808740	0.010629
8	2.808759	-0.945910	0.010590
1	0.000032	-0.000016	1.464539

Frequencies, cm^{-1}

-60.4432 92.5324 92.5329
96.6569 107.3061 350.9530
353.5384 353.5390 384.6951
447.0413 460.4196 484.9736
484.9751 561.7865 623.0380
623.0390 652.0539 734.9852
734.9866 1914.5365 1974.3881
1974.3930 1994.0426 2071.2594

TS$_{OSH}$

Charge = 0 Multiplicity = 2

Cartesian Coordinates

26	0.000012	-0.000003	-0.331401
6	-0.000007	1.359326	0.860733
6	-0.000007	-1.359311	0.860757
6	1.751608	-0.000007	-0.733459
6	-1.751578	-0.000007	-0.733483
8	2.872339	-0.000009	-1.036806
8	-0.000043	2.258681	1.597840
8	-0.000043	-2.258652	1.597881
8	-2.872306	-0.000009	-1.036844
1	0.000023	-0.000017	-1.887433

Frequencies, cm^{-1}

-490.8764 73.6951 79.4567
85.5812 95.0300 105.4588
317.8389 354.4739 364.0252
435.2951 450.1805 454.4935
485.5580 515.9610 535.9780
542.6469 562.1139 610.0055
664.3102 1827.1296 1973.2892
1986.2363 1989.4727 2058.0897

TS$_{OSL}$

Charge = 0 Multiplicity = 2

Cartesian Coordinates

26	0.132630	0.000359	-0.144593
6	-0.058038	-0.004449	1.659401
6	-0.965951	1.360206	-0.516274
6	-0.966771	-1.356815	-0.523547
6	1.960354	0.000518	-0.410575
8	-1.655690	-2.250352	-0.807539
8	-0.217860	-0.007520	2.810123
8	-1.654341	2.255656	-0.795481
8	3.086386	0.000902	-0.686033
1	0.266085	0.004408	-1.663160

Frequencies, cm^{-1}

-337.4393 72.7162 75.4490
93.0402 96.8229 141.6722
326.8808 333.9619 338.3500
410.8621 462.1880 482.2489
499.2472 525.2955 536.1859
554.3941 587.7086 661.8092
719.5485 1900.8645 1971.0948

1989.2327 1992.9728 2050.1103

TS_{BFI}

Charge = 0 Multiplicity = 2

Cartesian Coordinates

26	-0.000620	0.027481	-0.086659
6	-0.038918	1.749694	0.623871
6	0.035601	-1.583360	0.700312
6	1.717701	-0.017899	-0.576265
6	-1.715500	-0.094619	-0.575197
8	2.804423	-0.083113	-0.986732
8	-0.062816	2.820635	1.066443
8	0.058688	-2.610040	1.249482
8	-2.798476	-0.208333	-0.984993
1	0.008258	-0.390587	-1.536783

Frequencies, cm^{-1}

-421.1243 72.6195 75.0622
89.5681 92.7034 119.2074
316.9151 358.0102 379.5419
408.0109 465.7871 468.5998
494.2993 517.1361 536.0585
552.5862 572.9303 628.1067
676.0283 1930.6426 1965.9173
1978.2932 1989.3040 2051.8197

UB3LYP/ LanL2TZ(f) ECP[Fe]/ aug-cc-*p*VDZ [C,O,H] *(BS-I)*

Quasi-SP

Charge = 0 Multiplicity = 2

Cartesian Coordinates

26	0.000082	0.002786	-0.306823
6	-0.000970	1.487272	0.764963
6	0.000481	-1.294636	0.994261
6	1.764069	-0.036078	-0.765276
6	-1.763676	-0.037891	-0.766003
8	2.856903	-0.120792	-1.099123
8	-0.001669	2.418534	1.433838
8	0.000761	-2.139954	1.768376
8	-2.856302	-0.123691	-1.100250
1	0.000899	-1.057206	-1.412986

Frequencies, cm^{-1}

66.6773 80.6032 91.4446
95.0798 101.2368 297.4292
345.8129 357.7503 399.8700
419.6640 451.5214 460.6365
480.8571 516.7320 536.7642
580.4519 589.5223 674.8022
754.1921 1879.5253 2068.7308
2070.2919 2076.3309 2145.7802

EBT

Charge = 0 Multiplicity = 2

Cartesian Coordinates

26	-0.000004	0.000006	-0.024849
6	-0.009119	-1.752902	0.395394
6	0.009107	1.752935	0.395306
6	-1.786819	0.009125	-0.480128
6	1.786813	-0.009143	-0.480112
8	-2.900163	0.014534	-0.752124
8	-0.014808	-2.841685	0.758348
8	0.014796	2.841734	0.758212
8	2.900162	-0.014577	-0.752091
1	-0.000003	0.000050	1.492576

Frequencies, cm^{-1}

48.8331 91.5756 93.1941
96.3287 109.8384 328.2855
367.1826 386.3228 392.0007
437.8910 459.7552 472.7786
476.1667 538.3400 562.5344
591.2840 620.5324 668.6479
668.9916 1842.4342 2054.5324
2064.7381 2070.0468 2144.5907

TS_{mBPR}

Charge = 0 Multiplicity = 2

Cartesian Coordinates

26	0.000000	0.000003	-0.041346
6	1.784361	-0.370619	-0.042503
6	-1.784364	0.370613	-0.042523
6	-0.370614	-1.784360	-0.042481
6	0.370618	1.784365	-0.042546
8	-0.603731	-2.905162	0.019678
8	2.905163	-0.603744	0.019642
8	-2.905169	0.603723	0.019614
8	0.603736	2.905169	0.019576
1	-0.000004	0.000033	1.467228

Frequencies, cm^{-1}

-50.6625 97.9280 97.9281
99.9413 112.7273 356.5036
358.4912 358.4987 379.3526
415.7437 436.9549 466.0367
466.0379 575.8323 629.0189

629.0191 648.8352 730.9759
730.9842 1822.6459 2062.3193
2062.3221 2072.3614 2152.2074

TS_{OSH}

Charge = 0 Multiplicity = 2

Cartesian Coordinates

26 -0.000010 -0.000003 0.340414
6 0.000010 1.415029 -0.843960
6 0.000010 -1.415013 -0.843986
6 -1.776711 -0.000006 0.698687
6 1.776683 -0.000006 0.698724
8 -2.889184 -0.000008 0.971759
8 0.000039 2.330549 -1.534443
8 0.000039 -2.330522 -1.534485
8 2.889150 -0.000008 0.971819
1 -0.000024 -0.000018 1.895220

Frequencies, cm^{-1}

-444.6437 71.3226 81.5131
86.8718 96.6779 107.7689
305.1212 360.0314 360.4394
400.8993 432.1787 450.1363
457.3949 520.9123 527.0728
557.0092 576.6546 608.1596
691.8571 1819.2074 2060.1161
2073.8790 2078.1372 2147.7161

TS_{OSL}

Charge = 0 Multiplicity = 2

Cartesian Coordinates

26 0.122243 0.000389 -0.154294
6 0.060395 -0.004467 1.665359
6 0.976544 1.399424 -0.512176
6 0.977386 -1.396048 -0.519646
6 1.977762 0.000495 -0.403129
8 1.640336 -2.288415 -0.804487
8 0.209588 -0.007513 2.802169
8 1.638958 2.293697 -0.792246
8 3.089779 0.000858 -0.667492
1 0.233899 0.004424 -1.674358

Frequencies, cm^{-1}

-334.9497 71.9204 77.2034
94.1364 99.6727 138.8327
331.9160 332.9063 335.8538
383.2371 447.6260 452.3913
553.5479 588.3244 706.4226
764.8597 1896.8097 2055.8841
2069.7666 2085.2395 2141.7611

TS_{BFI}

Charge = 0 Multiplicity = 2

Cartesian Coordinates

26 -0.000785 0.022654 -0.087917
6 -0.039718 1.783379 0.599976
6 0.036536 -1.623565 0.698107
6 1.738064 -0.016396 -0.555915
6 -1.736401 -0.094097 -0.555052
8 2.816978 -0.072887 -0.944713
8 -0.063056 2.848935 1.013118
8 0.059888 -2.647494 1.216330
8 -2.811910 -0.198713 -0.943300
1 0.007456 -0.345070 -1.558931

Frequencies, cm^{-1}

-422.4329 70.6733 78.2627
92.7032 97.2579 121.5168
308.8870 354.7663 377.6276
383.6198 450.2207 465.2286
467.7932 503.2989 543.7080
571.1000 575.4699 653.3038
658.6135 1879.0579 2053.5229
2062.0476 2078.5862 2141.2651

UB3LYP/ 6-311+G(2d,p) *(BS-II)*

Quasi-SP

Charge = 0 Multiplicity = 2

Cartesian Coordinates

26 -0.023876 -0.290159 -0.012445
6 1.383987 0.909462 -0.010109
6 -1.423765 0.911196 -0.002879
6 -0.015964 -0.761963 1.760922
6 -0.024697 -0.744970 -1.789825
8 0.051083 -1.105815 2.843343
8 2.286688 1.602727 -0.008997
8 -2.289250 1.650704 0.002273
8 0.037089 -1.078873 -2.875719
1 0.961575 -1.465256 -0.021688

Frequencies, cm^{-1}

64.6841 77.7144 87.1066
90.0485 95.7063 284.4441
337.0082 347.3547 390.0192
408.3873 440.3727 447.8220

473.8726 503.9525 526.6561
747.1709 1892.7817 2085.0780
2085.8560 2091.7722 2160.5007

EBT

Charge = 0 Multiplicity = 2

Cartesian Coordinates

26	-0.000065	-0.252835	0.000000
6	1.797686	0.211012	0.000000
6	-1.797676	0.211534	0.000000
6	-0.000129	-0.685203	1.759352
6	-0.000129	-0.685203	-1.759352
8	-0.000185	-1.058009	2.836524
8	2.903746	0.479217	0.000000
8	-2.903656	0.480065	0.000000
8	-0.000185	-1.058009	-2.836524
1	-0.000293	-1.776526	0.000000

Frequencies, cm^{-1}

46.4058 86.9424 89.9798
92.0391 105.6183 318.5296
357.9022 375.2853 388.6423
424.3720 454.2967 465.4233
465.7808 516.0613 553.0133
580.4839 606.1385 654.9091
662.1264 1833.7176 2071.2977
2078.5412 2084.1434 2157.5741

TS_{mBPR}

Charge = 0 Multiplicity = 2

Cartesian Coordinates

26	-0.000095	-0.334111	0.000000
6	1.831180	-0.339759	0.000000
6	-1.831374	-0.339195	0.000000
6	-0.000088	-0.339386	1.831269
6	-0.000088	-0.339386	-1.831269
8	-0.000092	-0.413291	2.967362
8	2.967262	-0.413831	0.000000
8	-2.967478	-0.412912	0.000000
8	-0.000092	-0.413291	-2.967362
1	-0.000328	-1.852440	0.000000

Frequencies, cm^{-1}

-48.3320 94.3169 94.3233
96.7717 108.6290 345.8954
353.4360 353.4419 370.5155
410.9913 432.2864 460.1767
460.1899 567.4932 621.8980
621.9000 634.1539 717.4076
717.4198 1791.0908 2078.0191
2078.0194 2088.0973 2166.1564

TS_{OSH}

Charge = 0 Multiplicity = 2

Cartesian Coordinates

26	-0.000086	-0.345380	0.000000
6	1.421032	0.851397	0.000000
6	-1.420831	0.851842	0.000000
6	-0.000141	-0.705194	1.786937
6	-0.000141	-0.705194	-1.786937
8	-0.000184	-0.976543	2.891611
8	2.330959	1.535856	0.000000
8	-2.330547	1.536580	0.000000
8	-0.000184	-0.976543	-2.891611
1	-0.000337	-1.905576	0.000000

Frequencies, cm^{-1}

-443.8016 70.2927 79.0364
83.6499 92.2336 103.1009
306.6370 351.1147 352.7344
390.4547 421.7413 437.2125
448.4587 511.3222 515.5509
548.3676 564.0527 595.9056
687.0361 1824.9193 2076.2812
2089.3352 2093.6070 2162.2411

TS_{OSL}

Charge = 0 Multiplicity = 2

Cartesian Coordinates

26	0.124106	0.000382	-0.154618
6	-0.059721	-0.004505	1.678047
6	-0.985617	1.404469	-0.513552
6	-0.986461	-1.401087	-0.521077
6	1.996879	0.000485	-0.402562
8	-1.647878	-2.283527	-0.808671
8	-0.211335	-0.007486	2.806425
8	-1.646504	2.288832	-0.796415
8	3.099735	0.000865	-0.669911
1	0.230612	0.004418	-1.676500

Frequencies, cm^{-1}

-298.6011 71.0431 75.6804
91.1203 95.2060 136.4060
325.9076 327.7245 329.0537
373.3945 438.6189 441.2246
460.8494 510.8859 543.5615
545.4262 575.8688 710.0587
766.1374 1915.5012 2070.5022

2084.6289 2102.3050 2156.9443

TS_{BFI}

Charge = 0 Multiplicity = 2

Cartesian Coordinates

26	0.000052	0.024458	-0.081978
6	0.000207	1.800979	0.610306
6	0.000134	-1.636062	0.702373
6	1.745368	-0.054657	-0.558048
6	-1.745424	-0.054567	-0.557471
8	2.811841	-0.138847	-0.952724
8	0.000288	2.860203	1.017827
8	0.000176	-2.657026	1.209285
8	-2.812031	-0.138702	-0.951794
1	-0.000205	-0.345059	-1.557656

Frequencies, cm^{-1}

-401.9689 69.2474 76.1941
89.6379 93.8461 115.7948
297.0127 347.2106 367.3692
380.6853 440.8454 457.8011
460.7885 495.2782 534.5544
561.8362 567.5326 647.1821
659.4143 1878.9720 2067.8493
2076.3958 2095.5005 2155.6685

UMP2/ LanL2TZ(f) ECP[Fe]/ aug-cc-*p*VDZ [C,O,H] *(BS-I)*

Quasi-SP

Charge = 0 Multiplicity = 2

Cartesian Coordinates

26	0.000000	-0.065507	-0.321489
6	0.000011	1.485459	0.441602
6	-0.000011	-0.939180	1.114182
6	1.657447	-0.235040	-0.744201
6	-1.657446	-0.235013	-0.744212
8	2.788708	-0.365988	-0.978603
8	0.000018	2.534018	0.952633
8	-0.000018	-1.496443	2.144812
8	-2.788708	-0.365942	-0.978621
1	-0.000006	-1.208363	-1.113024

Frequencies, cm^{-1}

86.3222 100.7886 107.8092
109.8086 115.2651 405.0044
470.8867 496.4196 519.2793
565.9236 569.4428 626.7223
634.8723 664.2465 695.0544
752.1245 821.6941 895.0807
1165.5376 2012.0757 2036.6218
2062.2964 2150.5737 2560.0536

EBT

Charge = 0 Multiplicity = 2

Cartesian Coordinates

26	0.000015	-0.000174	0.005477
6	0.110082	-1.570059	0.618652
6	-0.110093	1.570099	0.617986
6	-1.597211	-0.111720	-0.648747
6	1.597219	0.111822	-0.648721
8	-2.662627	-0.186052	-1.123358
8	0.185581	-2.645801	1.048785
8	-0.185600	2.645806	1.048385
8	2.662599	0.186627	-1.123337
1	0.000037	-0.000516	1.358988

Frequencies, cm^{-1}

95.6748 98.6789 99.5561
108.8520 139.7546 353.2056
439.2769 454.8167 505.7318
545.3488 565.1078 569.5012
630.0876 654.9483 740.1582
751.9260 846.3033 885.0426
1166.0647 1996.7652 2020.7196
2148.9099 2317.7733 2796.1293

TS_{mBPR}

Charge = 0 Multiplicity = 2

Cartesian Coordinates

26	-0.000010	0.000008	-0.101850
6	1.335590	-1.062380	-0.038454
6	-1.335613	1.062381	-0.038424
6	-1.062365	-1.335609	-0.038406
6	1.062396	1.335594	-0.038472
8	-1.783761	-2.242598	0.071337
8	2.242556	-1.783808	0.071258
8	-2.242575	1.783812	0.071304
8	1.783858	2.242535	0.071226
1	0.000014	0.000024	1.267362

Frequencies, cm^{-1}

-182.8092 103.3793 103.3797
117.5562 118.5660 307.7573
449.2607 449.2610 495.0303
527.3609 527.3610 552.9617

587.0342 633.6327 732.8709
732.8710 759.3112 974.2097
974.2098 1994.8022 1994.8024
2041.1749 2058.0741 2577.3389

TS_{OSH}

Charge = 0 Multiplicity = 2

Cartesian Coordinates

26	-0.000112	-0.000018	-0.298914
6	0.000435	1.218553	0.826251
6	0.000432	-1.218471	0.826377
6	1.625665	-0.000041	-0.764173
6	-1.626332	-0.000037	-0.762655
8	2.751878	-0.000056	-1.057418
8	0.000814	2.083714	1.607691
8	0.000809	-2.083551	1.607907
8	-2.752809	-0.000049	-1.054874
1	-0.000768	-0.000092	-1.748576

Frequencies, cm^{-1}

-905.0678 81.4743 99.7134
106.5698 108.7806 137.3108
475.7347 505.3135 520.4134
597.9026 611.6408 612.2310
618.9537 637.4645 718.3225
730.5852 781.1195 911.2460
1072.5826 1990.8506 2012.5896
2100.9556 2117.3003 2407.4735

TS_{OSL}

Charge = 0 Multiplicity = 2

Cartesian Coordinates

26	-0.115176	-0.000618	-0.232134
6	-0.118637	0.003367	1.482946
6	0.988488	-1.259979	-0.433024
6	0.983403	1.262219	-0.438871
6	-1.871714	-0.004702	-0.529286
8	1.755750	2.131173	-0.538834
8	-0.109667	0.006119	2.651948
8	1.764360	-2.126244	-0.528954
8	-3.019356	-0.007306	-0.690348
1	-0.077571	-0.003710	-1.615998

Frequencies, cm^{-1}

-123.1192 101.2046 103.1714
115.8882 126.3228 330.4814
420.5241 461.0838 494.5005
537.7607 561.9042 576.2962
611.0665 672.9236 674.9627
731.7641 850.6324 860.3467
1455.3673 2014.9745 2028.5670
2084.4289 2485.9794 3318.4634

TS_{BFI}

Charge = 0 Multiplicity = 2

Cartesian Coordinates

26	0.000548	0.012511	-0.092001
6	0.000049	1.619021	0.686267
6	-0.000292	-1.468384	0.719123
6	1.575802	-0.034401	-0.698170
6	-1.574956	-0.034392	-0.697223
8	2.664160	-0.078595	-1.109018
8	-0.000321	2.677429	1.164614
8	-0.000895	-2.448043	1.364067
8	-2.663577	-0.078593	-1.107382
1	-0.000114	-0.505834	-1.350156

Frequencies, cm^{-1}

-600.1872 49.2760 92.1618
111.2524 137.3387 161.0617
432.8124 447.5158 497.8826
532.3572 533.2676 601.7420
645.0872 693.8238 706.8349
746.0694 916.7356 1016.0012
1145.9248 2004.9793 2059.2284
2114.2966 2188.5440 2732.9509

Table S2. Geometries and Frequencies of $HM(CO)_4$ Complexes Calculated at B3LYP/LanL2TZ(f) [Fe]/ 6-31G(d,p)[C,O,H] Level (BS-III) Presented in Table 2.

$[HCo(CO)_4]^+$ cation-rad

Quasi-SP

Charge = 1 Multiplicity = 2

Cartesian Coordinates

6	0.007867	-1.484053	0.921891
6	0.000965	1.438963	0.968727
6	-1.825009	-0.016374	-0.786647
6	1.817366	-0.008064	-0.803651
8	-2.909743	0.035399	-1.104528
8	0.014370	-2.341178	1.659461
8	0.001286	2.333985	1.659511
8	2.898688	0.049409	-1.132074

1 -0.007275 0.964082 -1.415663
27 -0.001358 -0.043253 -0.335008

Frequencies, cm^{-1}

69.4400 77.5265 92.2824
93.1962 100.2916 263.4066
278.6631 289.3875 329.2224
332.7193 364.6907 375.5773
429.4661 446.3922 459.0390
497.9547 500.1029 715.1711
821.0577 1956.3725 2242.4727
2251.7009 2255.6180 2279.6716

EBT

Charge = 1 Multiplicity = 2

Cartesian Coordinates

6 1.871911 0.010501 0.617210
6 -1.871745 -0.010439 0.617640
6 0.009942 -1.778454 -0.524264
6 -0.010038 1.778447 -0.524442
8 0.015916 -2.856570 -0.874537
8 2.947306 0.016210 0.964201
8 -2.947180 -0.016262 0.964507
8 -0.016161 2.856525 -0.874828
1 0.000043 -0.000056 -1.513551
27 0.000018 0.000019 -0.038447

Frequencies, cm^{-1}

50.4374 81.5608 85.2741
85.3200 106.5459 165.1190
237.5437 271.6441 299.1417
339.7841 345.2202 370.0780
379.0744 393.8018 453.0628
481.5475 514.7015 526.2845
649.4514 1891.9023 2228.2152
2245.7036 2250.3858 2271.5159

TS$_{mBPR}$

Charge = 1 Multiplicity = 2

Cartesian Coordinates

7 -0.000007 0.000011 -0.032923
6 0.276593 1.839890 -0.050394
6 -0.276572 -1.839872 -0.050520
6 1.839874 -0.276585 -0.050373
6 -1.839887 0.276581 -0.050539
8 2.957526 -0.444778 0.019583
8 0.444770 2.957546 0.019551
8 -0.444715 -2.957519 0.019654
8 -2.957536 0.444722 0.019622
1 -0.000222 -0.000130 1.472607

Frequencies, cm^{-1}

-250.1640 100.9219 103.4045
103.4046 115.0687 125.6749
335.6026 335.6029 339.7511
362.7000 384.7938 393.1443
393.1446 525.0457 539.4130
563.4648 563.4652 680.5803
680.5807 1721.9758 2228.9679
2228.9680 2238.5200 2274.4795

TS$_{OSH}$

Charge = 1 Multiplicity = 2

Cartesian Coordinates

6 -0.000042 1.441820 0.952826
6 -0.000047 -1.441784 0.952880
6 1.807328 -0.000017 -0.772071
6 -1.807257 -0.000012 -0.772240
8 2.907738 -0.000024 -1.038251
8 -0.000071 2.328518 1.654620
8 -0.000078 -2.328456 1.654708
8 -2.907642 -0.000015 -1.038524
1 0.000085 -0.000036 -1.895655
27 0.000017 -0.000007 -0.375301

Frequencies, cm^{-1}

-728.4388 75.7076 79.2146
91.4100 95.1661 109.4016
250.0072 295.3918 305.6936
318.6550 324.2303 369.6312
392.9750 440.2895 443.8275
485.2562 488.7048 521.0749
675.1626 1818.9319 2238.7028
2249.9614 2252.3076 2277.4496

TS$_{OSL}$

Charge = 1 Multiplicity = 2

Cartesian Coordinates

6 -0.062061 -0.000208 1.726684
6 1.068868 -1.452181 -0.479957
6 1.068827 1.452327 -0.479608
6 -2.039187 0.000036 -0.522256
8 1.746876 2.328292 -0.712990
8 -0.012577 -0.000358 2.856785
8 1.746941 -2.328070 -0.713550
8 -3.131099 0.000056 -0.811279
1 -0.121550 0.000205 -1.646282
27 -0.107343 0.000022 -0.176838

Frequencies, cm^{-1}

-297.5944 74.5934 79.1869
93.7743 99.1028 140.6010
286.2935 290.7915 307.0726
307.3215 327.9250 360.3789
373.7716 449.1269 459.3603
476.0763 493.5859 766.1428
845.9635 1981.5106 2236.3447
2248.9993 2259.7974 2277.9186

TS_{BFI}

Charge = 1 Multiplicity = 2

Cartesian Coordinates

27	0.003849	-0.000002	-0.064282
6	1.882611	-0.000678	0.634417
6	-1.831179	0.000636	0.665199
6	-0.014560	-1.778893	-0.551534
6	-0.013293	1.778912	-0.551495
8	-0.033188	-2.856108	-0.904404
8	2.948128	-0.001029	1.009287
8	-2.890722	0.001004	1.059497
8	-0.031157	2.856147	-0.904342
1	-0.189896	0.000083	-1.524220

Frequencies, cm^{-1}

-183.2187 70.5641 83.3238
86.3920 101.5636 119.8841
238.7531 262.9278 299.0911
313.4731 341.7236 379.5518
391.2690 406.2869 450.3319
477.9620 512.8742 524.2110
652.8944 1904.2198 2227.4737
2243.1630 2253.2207 2271.6265

$HFe(CO)_4$ radical

Quasi-SP

Charge = 0 Multiplicity = 2

Cartesian Coordinates

26	-0.006611	-0.009225	-0.265250
6	-0.001593	-1.404245	0.907154
6	-0.005645	1.373555	0.931139
6	-1.766793	-0.021350	-0.732700
6	1.751439	-0.016680	-0.740885
8	-2.860751	0.024568	-1.077504
8	0.002592	-2.285219	1.644213
8	-0.005100	2.280707	1.634961
8	2.843372	0.032580	-1.091591
1	-0.009859	0.999956	-1.417344

Frequencies, cm^{-1}

68.0480 82.7511 93.4196
97.1487 103.7794 310.2420
346.6275 363.1107 402.7738
428.8517 457.9388 461.1624
486.9833 520.9959 547.9227
590.4732 593.8184 699.6557
784.3553 1880.8653 2107.6187
2111.6781 2116.3567 2178.6741

EBT

Charge = 0 Multiplicity = 2

Cartesian Coordinates

26	-0.000223	-0.000103	-0.014160
6	-0.016365	1.741536	0.433119
6	0.015615	-1.741778	0.433001
6	1.773661	0.016376	-0.507579
6	-1.773767	-0.016483	-0.508789
8	2.880806	0.027041	-0.811598
8	-0.026426	2.827006	0.812231
8	0.025377	-2.827242	0.812144
8	-2.880648	-0.027026	-0.813772
1	-0.000741	-0.000235	1.503103

Frequencies, cm^{-1}

52.1227 91.7354 93.9796
96.1363 111.3905 323.6540
365.7682 388.4928 401.7433
430.2086 463.7844 475.9051
483.4111 513.2077 567.2086
593.1565 618.1041 658.1203
662.7024 1821.7044 2094.8181
2105.4430 2109.5771 2175.7813

TS_{mBPR}

Charge = 0 Multiplicity = 2

Cartesian Coordinates

26	-0.000001	-0.000037	-0.038490
6	1.093655	-1.459283	-0.042489
6	-1.093666	1.459203	-0.042502
6	-1.459251	-1.093703	-0.042590
6	1.459248	1.093632	-0.042643
8	-2.376803	-1.781799	0.019897
8	1.781734	-2.376840	0.020035
8	-1.781756	2.376757	0.020000

8 2.376801 1.781732 0.019785
1 0.000014 -0.000018 1.467862

Frequencies, cm^{-1}

-65.7520 98.4436 98.4507
100.1842 113.2561 334.5877
359.6445 359.6448 374.8239
412.1071 433.0700 458.9749
458.9869 576.1556 627.1593
627.1619 630.7520 740.5076
740.5166 1775.2837 2101.1023
2101.1212 2110.7653 2182.3973

TS_{OSH}

Charge = 0 Multiplicity = 2

Cartesian Coordinates

26 0.000037 -0.000147 -0.338082
6 0.000084 -1.406995 0.843010
6 -0.000345 1.407687 0.841836
6 -1.772566 -0.000562 -0.702696
6 1.772747 -0.000019 -0.702171
8 -2.886727 -0.000839 -0.978570
8 0.000146 -2.324018 1.535016
8 -0.000558 2.325286 1.533077
8 2.886990 0.000045 -0.977715
1 0.000261 -0.000806 -1.892808

Frequencies, cm^{-1}

-465.5721 74.9535 84.6096
87.4321 100.2837 110.9902
311.0869 363.1478 366.6586
404.7570 437.9198 449.5733
459.5043 529.4611 532.1587
559.2450 582.5463 610.3565
695.8868 1802.8527 2101.5699
2111.7352 2117.1483 2178.9219

TS_{OSL}

Charge = 0 Multiplicity = 2

Cartesian Coordinates

26 -0.073003 -0.168041 -0.508665
6 0.018646 1.311577 0.546719
6 0.162926 -1.410444 0.785859
6 1.593915 -0.076877 -1.207093
6 -1.656966 0.099107 -1.466963
8 2.631182 -0.052079 -1.701920
8 0.103609 2.234607 1.225178
8 0.280868 -2.242006 1.570694
8 -2.606066 0.163891 -2.104836
1 -0.115303 -1.420864 -1.377788

Frequencies, cm^{-1}

-333.3162 74.3356 80.1866
97.3596 102.9099 140.9630
336.2700 341.3464 344.6080
384.2402 454.5580 460.0685
474.5070 527.7419 562.6411
562.7410 598.7269 733.2257
798.6824 1897.2077 2098.5248
2110.4957 2122.0007 2174.7041

TS_{BFI}

Charge = 0 Multiplicity = 2

Cartesian Coordinates

26 -0.000014 0.020398 -0.083170
6 -0.001292 1.771205 0.608136
6 0.001169 -1.629025 0.687782
6 1.732348 -0.045897 -0.558971
6 -1.732259 -0.048411 -0.559038
8 2.811042 -0.115697 -0.951890
8 -0.002062 2.834424 1.034904
8 0.001891 -2.658227 1.200095
8 -2.810837 -0.119774 -0.951996
1 0.000290 -0.343381 -1.553925

Frequencies, cm^{-1}

-405.8646 72.6776 80.9818
94.6905 97.9205 123.4379
310.3148 357.4550 380.8366
390.5930 452.9132 467.8474
469.1837 508.7596 551.1174
576.6473 580.8332 654.4841
663.0514 1860.6352 2095.8994
2103.1288 2115.5695 2173.3239

$[HMn(CO)_4]^-$ anion- radical

Quasi-SP

Charge = -1 Multiplicity = 2

Cartesian Coordinates

6 0.000384 1.491015 0.647568
6 -0.000298 -1.199333 1.045850
6 1.762187 -0.083174 -0.754389
6 -1.762203 -0.082343 -0.754335
8 2.879511 -0.173628 -1.085218
8 0.000633 2.488047 1.262604

8 -0.000608 -1.974751 1.923322
8 -2.879577 -0.172252 -1.085147
1 -0.000269 -1.155945 -1.457420
25 0.000007 -0.037615 -0.311009

Frequencies, cm^{-1}

65.0216 81.1124 82.5433
94.9032 99.1078 286.0716
368.2457 390.7692 442.2408
456.3806 489.9244 506.3028
512.2743 571.0951 576.9788
617.9842 647.8636 703.3766
730.8165 1720.7957 1960.1271
1967.9448 1972.5344 2056.7840

EBT

Charge = -1 Multiplicity = 2

Cartesian Coordinates

6 -0.000026 -1.726595 0.583601
6 -0.000033 1.726596 0.583599
6 -1.724080 -0.000004 -0.514696
6 1.724692 0.000003 -0.514598
8 -2.799601 -0.000006 -0.976726
8 -0.000958 -2.812174 1.022894
8 -0.000968 2.812175 1.022890
8 2.800311 0.000004 -0.976369
1 0.000129 -0.000001 -1.561624
25 0.000251 0.000000 -0.000293

Frequencies, cm^{-1}

59.0524 80.2593 81.4811
88.4520 101.5551 350.1425
394.9989 418.5764 440.4216
445.8090 499.7483 504.6504
510.1898 518.6870 589.9505
601.3325 658.8628 666.3022
715.8117 1767.5227 1943.7304
1963.4116 1969.4162 2053.8990

TS_{mBPR}

Charge = -1 Multiplicity = 2

Cartesian Coordinates

25 -0.000006 -0.000001 -0.019924
6 1.003307 1.524085 -0.037094
6 -1.003306 -1.524097 -0.037111
6 1.524090 -1.003302 -0.037071
6 -1.524093 1.003311 -0.037135
8 2.500388 -1.645997 -0.004447
8 1.646019 2.500374 -0.004484
8 -1.646002 -2.500395 -0.004513
8 -2.500381 1.646023 -0.004552
1 -0.000035 0.000007 1.532533

Frequencies, cm^{-1}

-64.2653 84.2308 84.2311
95.2880 98.7065 354.3502
354.3505 393.7494 422.8184
438.1595 460.7438 498.1306
498.1307 591.4003 642.6498
642.6498 691.2180 756.3984
756.3984 1727.1576 1959.7542
1959.7547 1975.5014 2063.6753

TS_{OSH}

Charge = -1 Multiplicity = 2

Cartesian Coordinates

6 0.000053 -1.383361 0.806021
6 0.000048 1.383363 0.806018
6 -1.781716 -0.000004 -0.689740
6 1.781626 0.000003 -0.689973
8 -2.915948 -0.000006 -0.967873
8 0.000105 -2.297531 1.539707
8 0.000097 2.297534 1.539703
8 2.915822 0.000004 -0.968256
1 -0.000126 -0.000003 -1.955817
25 -0.000022 0.000000 -0.343375

Frequencies, cm^{-1}

-280.6900 72.6428 76.3306
84.2247 98.7804 103.9180
372.2450 380.0726 395.0678
448.2366 488.4532 493.1989
496.9976 564.9674 577.0403
616.1203 618.5475 680.0317
698.4240 1675.0156 1951.4125
1972.0052 1973.9407 2056.8114

TS_{OSL}

Charge = -1 Multiplicity = 2

Cartesian Coordinates

6 -0.084196 -0.000033 1.612738
6 -0.962337 1.378851 -0.523546
6 -0.962338 -1.378828 -0.523602
6 1.974833 0.000006 -0.356364
8 -1.638601 -2.296081 -0.794212
8 -0.254949 -0.000058 2.771519
8 -1.638599 2.296117 -0.794116
8 3.123939 0.000010 -0.562257

1	0.274378	0.000026	-1.767079
25	0.127821	0.000004	-0.178230

Frequencies, cm^{-1}

-292.8484	64.9256	79.5026
91.2298	98.6845	151.1285
340.8643	345.7747	377.5434
432.0123	491.4017	513.5292
523.4545	562.3262	599.5688
608.9842	641.0250	673.8090
747.4743	1735.1713	1956.0377
1962.2346	1974.6826	2051.5162

TS_{BFI}

Charge = -1 Multiplicity = 2

Cartesian Coordinates

25	-0.000012	0.046834	-0.094690
6	-0.000650	1.764493	0.598467
6	0.000494	-1.539170	0.745789
6	1.723604	-0.091642	-0.595491
6	-1.723476	-0.092832	-0.595687
8	2.801568	-0.219172	-1.033841
8	-0.001049	2.827103	1.084958
8	0.000821	-2.533945	1.368446
8	-2.801303	-0.221105	-1.034157
1	0.000177	-0.238995	-1.634458

Frequencies, cm^{-1}

-416.4530	70.3833	71.8266
91.5125	92.2601	123.2542
345.9636	390.1869	410.9753
435.8541	460.2697	501.6662
508.3719	516.3111	579.5925
596.6816	616.1231	655.0111
724.4579	1769.0405	1945.0091
1965.4975	1968.7652	2050.9134

$HRu(CO)_4$ radical

Quasi-SP

Charge = 0 Multiplicity = 2

Cartesian Coordinates

44	0.000125	-0.033658	-0.303233
6	-0.002340	1.667705	0.776992
6	0.001370	-1.319737	1.139214
6	1.933509	-0.068860	-0.700163
6	-1.932957	-0.073656	-0.701175
8	3.048955	-0.166574	-0.954671
8	-0.003776	2.640551	1.384892
8	0.002236	-2.107126	1.977230
8	-3.048040	-0.174102	-0.956213
1	0.001994	-1.293761	-1.356854

Frequencies, cm^{-1}

66.1846	76.4920	84.0686
85.4979	88.0250	282.7992
319.5381	331.0357	379.1307
396.0841	417.1568	419.2673
459.6503	463.2900	492.8331
496.5159	544.5537	725.0717
806.2231	1935.9864	2097.5360
2099.9096	2115.0465	2171.5152

SP

Charge = 0 Multiplicity = 2

Cartesian Coordinates

44	-0.000037	-0.000007	-0.003494
6	-0.422976	1.923186	-0.035998
6	0.423025	-1.923173	-0.036000
6	1.923177	0.422886	-0.044438
6	-1.923248	-0.422903	-0.044424
8	3.045635	0.669366	-0.023127
8	-0.669650	3.045367	-0.006435
8	0.669945	-3.045301	-0.006436
8	-3.045706	-0.669388	-0.023187
1	-0.000034	-0.000010	1.592353

Frequencies, cm^{-1}

40.7677	88.5637	88.5964
91.4161	97.1579	349.5710
353.1429	353.5042	387.0087
387.3309	387.4550	436.6643
442.9473	511.2052	584.4398
584.5754	612.8624	728.8229
730.3296	1976.0142	2090.2826
2090.6919	2100.1618	2181.7865

TS_{OSH}

Charge = 0 Multiplicity = 2

Cartesian Coordinates

44	0.000083	-0.000135	-0.327973
6	0.000090	-1.458512	0.989886
6	-0.000352	1.459328	0.988672
6	-1.937463	-0.000571	-0.671089
6	1.937706	0.000015	-0.670617
8	-3.057231	-0.000842	-0.918939

8 -0.000034 -2.356375 1.708560
8 -0.000747 2.357787 1.706601
8 3.057535 0.000082 -0.918195
1 0.000290 -0.000831 -2.014501

Frequencies, cm^{-1}

-622.9631 72.1226 77.3597
79.5790 87.2012 95.7327
294.4713 327.7570 329.1120
395.6521 399.0970 407.8811
441.7169 467.2839 484.3950
490.4278 512.5324 559.8799
694.8221 1802.1826 2083.9345
2104.7085 2112.0560 2174.4988

TS$_{OSL}$

Charge = 0 Multiplicity = 2

Cartesian Coordinates

44 0.121643 0.000011 -0.176617
6 -0.037866 -0.000197 1.830541
6 -1.133294 1.426055 -0.521524
6 -1.133358 -1.425893 -0.521912
6 2.154108 -0.000006 -0.348144
8 -1.858344 -2.283538 -0.773987
8 -0.155755 -0.000313 2.971290
8 -1.858271 2.283778 -0.773356
8 3.282283 0.000010 -0.555562
1 0.270852 0.000266 -1.809688

Frequencies, cm^{-1}

-226.9827 69.5945 73.8795
86.7519 91.1742 151.6720
303.5481 304.1616 329.0778
364.3132 383.2937 435.9622
441.1800 472.8172 488.4920
514.4846 526.2805 743.5420
818.5857 1946.2193 2082.7445
2102.8899 2109.4732 2165.4515

TS$_{BFI}$

Charge = 0 Multiplicity = 2

Cartesian Coordinates

44 0.000004 -0.005444 -0.139713
6 -0.001024 1.922165 0.582758
6 0.000933 -1.770512 0.615467
6 1.943424 -0.024946 -0.423215
6 -1.943390 -0.027008 -0.423247
8 3.066009 -0.073947 -0.656820
8 -0.001605 3.014711 0.937852
8 0.001489 -2.825138 1.081777
8 -3.065918 -0.077199 -0.656871
1 0.000367 -0.666071 -1.610726

Frequencies, cm^{-1}

-331.7625 71.4728 74.5165
86.0344 91.0813 114.1980
297.2255 316.3553 353.5473
356.4380 394.7055 441.4193
444.4357 474.9329 522.9866
532.5277 552.4514 687.3630
759.2138 1962.9193 2081.5569
2090.9653 2103.8138 2173.3983

$HOs(CO)_4$ radical

Quasi-SP

Charge = 0 Multiplicity = 2

Cartesian Coordinates

6 -0.007631 -1.509883 1.037974
6 -0.001095 1.440380 1.023684
6 -1.923904 -0.024409 -0.612800
6 1.928825 -0.032838 -0.596846
8 -3.041824 -0.005274 -0.885902
8 -0.012708 -2.407863 1.757858
8 -0.002484 2.301482 1.791715
8 3.049062 -0.018691 -0.860601
1 0.008645 1.097034 -1.510854
76 0.001024 0.009293 -0.237181

Frequencies, cm^{-1}

73.7911 85.5891 86.2103
91.5780 93.9410 335.2394
364.8970 386.6812 431.1966
431.4057 478.0731 490.0431
496.0861 500.3302 528.9445
531.8428 581.9564 712.1289
833.5888 2019.4839 2088.7333
2091.2180 2114.6227 2173.2487

SP

Charge = 0 Multiplicity = 2

Cartesian Coordinates

76 -0.000011 0.000007 0.019111
6 1.751058 0.885772 -0.038642
6 -1.751043 -0.885809 -0.038879
6 0.885722 -1.750849 -0.044595

6	-0.885704	1.750881	-0.044578
8	1.405833	-2.778565	-0.070774
8	2.778919	1.405744	-0.059499
8	-2.778875	-1.405845	-0.059583
8	-1.405775	2.778617	-0.070756
1	-0.000187	-0.000088	1.632609

Frequencies, cm^{-1}

40.1579	89.9208	89.9305
92.9318	97.9492	383.3336
385.2793	385.3652	415.9746
416.0626	477.5433	485.0848
494.3839	532.5846	607.0907
607.1616	628.9579	749.3773
750.8912	2080.0897	2080.3647
2096.7175	2153.8683	2190.9361

TS$_{OSH}$

Charge = 0 Multiplicity = 2

Cartesian Coordinates

6	0.000073	-1.433647	1.038172
6	-0.000361	1.434489	1.036984
6	-1.938737	-0.000506	-0.555817
6	1.938914	0.000079	-0.555288
8	-3.065498	-0.000745	-0.784856
8	0.000026	-2.325610	1.770853
8	-0.000679	2.327067	1.768914
8	3.065740	0.000180	-0.784014
1	0.000274	-0.000830	-1.956161
76	0.000049	-0.000116	-0.257833

Frequencies, cm^{-1}

-555.4435	81.2454	82.3276
89.2603	97.2650	99.9804
365.3315	372.9889	383.8884
433.9668	470.6701	481.3299
491.6917	512.1984	528.3136
532.2548	541.2292	592.8106
762.5412	1955.5784	2071.9091
2093.7348	2100.0546	2172.3065

TS$_{OSL}$

Charge = 0 Multiplicity = 2

Cartesian Coordinates

76	0.093936	0.000202	-0.153623
6	-0.053943	-0.000421	1.824800
6	-1.170325	1.409010	-0.458435
6	-1.169888	-1.408903	-0.458473
6	2.087821	0.000088	-0.272604
8	-1.885304	-2.289069	-0.671146
8	-0.154373	-0.000783	2.970642
8	-1.885898	2.288997	-0.671280
8	3.230895	-0.000972	-0.417845
1	0.256326	0.000625	-1.819326

Frequencies, cm^{-1}

-210.3816	77.5211	82.9249
91.2628	98.2593	187.7827
345.5270	348.0925	384.5258
428.1662	432.9133	492.8865
493.8339	514.4905	528.2246
548.0803	561.0562	738.8950
817.9348	2020.2504	2077.3066
2079.0974	2108.0605	2166.3343

TS$_{BFI}$

Charge = 0 Multiplicity = 2

Cartesian Coordinates

76	-0.000003	-0.000957	-0.123590
6	-0.000962	1.870245	0.614688
6	0.000898	-1.743164	0.650761
6	1.942423	-0.026575	-0.381659
6	-1.942390	-0.028709	-0.381657
8	3.069410	-0.069669	-0.605516
8	-0.001450	2.949169	1.022909
8	0.001369	-2.768225	1.188372
8	-3.069328	-0.073044	-0.605518
1	0.000367	-0.663907	-1.621939

Frequencies, cm^{-1}

-355.3572	73.6396	82.8138
88.0009	98.6210	126.6041
350.9628	361.9240	393.3972
417.9485	426.9552	491.4134
502.4760	508.2615	552.3531
556.8408	582.0275	705.1918
795.1908	2059.5373	2075.9145
2096.5527	2109.2165	2175.3688

$HMn(CO)_4$ molecule

Quasi-SP

Charge = 0 Multiplicity = 1

Cartesian Coordinates

6	-0.005588	-1.454958	0.853035
6	0.000034	1.217437	0.789356
6	-1.818435	0.075133	-0.703475

6 1.823241 0.070212 -0.691179
8 -2.928212 0.255493 -0.941022
8 -0.010504 -2.291941 1.644165
8 -0.000403 2.096495 1.534744
8 2.935264 0.252419 -0.916457
1 0.006571 0.903701 -1.629217
25 0.001150 -0.114015 -0.417146

Frequencies, cm^{-1}

72.5988 85.3505 93.7433
104.1777 107.0174 330.7283
363.9309 366.9225 415.8678
454.7496 462.6067 488.4348
541.7969 566.4113 579.1612
632.5847 635.9638 713.8100
768.2504 1807.7766 2082.3300
2094.0241 2095.3478 2169.0381

SP

Charge = 0 Multiplicity = 1

Cartesian Coordinates

6 -0.012601 -1.844222 0.008329
6 0.007246 1.838335 -0.072316
6 -1.842645 0.014299 0.109982
6 1.832977 -0.013950 -0.211617
8 -2.953609 0.033851 0.408581
8 0.002071 -2.969608 0.248420
8 0.031772 2.973557 0.113532
8 2.979133 -0.012303 -0.110981
1 0.112766 0.022441 1.292813
25 -0.019902 -0.007728 -0.223020

Frequencies, cm^{-1}

23.1577 92.5243 92.9595
99.1361 114.2543 346.0267
348.2943 374.8868 416.7433
418.1208 464.9400 466.9728
508.0066 584.1391 639.0742
639.4930 671.7391 803.9425
805.2343 1903.8306 2078.6563
2079.0742 2101.0357 2173.1780

TS_{OSH}

Charge = 0 Multiplicity = 1

Cartesian Coordinates

6 0.000102 -1.284890 0.844720
6 -0.000285 1.285583 0.843667
6 -1.834912 -0.000561 -0.697999
6 1.835128 -0.000012 -0.697543
8 -2.966281 -0.000812 -0.890938
8 0.000037 -2.166463 1.587791
8 -0.000617 2.167769 1.586010
8 2.966554 0.000075 -0.890165
1 0.000378 -0.000850 -2.053090
25 0.000075 -0.000177 -0.433823

Frequencies, cm^{-1}

-380.9771 82.9870 87.2574
103.3166 105.7387 116.4136
364.5258 366.3560 366.9892
415.8678 439.8789 466.0843
502.5818 548.2949 580.6294
594.6106 635.5723 686.2438
720.2734 1727.4729 2078.1689
2090.9796 2104.9654 2171.8340

TS_{OSL}

Charge = 0 Multiplicity = 1

Cartesian Coordinates

6 -0.134694 -0.004448 1.724589
6 -0.994047 1.283998 -0.505613
6 -0.994853 -1.280667 -0.512286
6 2.115255 0.000582 -0.480534
8 -1.701757 -2.143672 -0.808104
8 -0.409263 -0.007421 2.843474
8 -1.700374 2.149023 -0.796872
8 3.175331 0.001336 -0.910168
1 0.358281 0.004059 -1.660046
25 0.191210 0.000201 -0.092941

Frequencies, cm^{-1}

-336.0439 71.2172 80.6102
101.8544 108.1171 128.8965
335.0062 340.8327 360.4301
370.4718 442.4400 485.4284
511.8904 530.2632 553.1416
621.0926 660.0526 703.2108
780.9770 1805.0246 2070.5625
2075.7836 2111.6160 2165.1328

TS_{BFI}

Charge = 0 Multiplicity = 1

Cartesian Coordinates

25 -0.000059 0.063241 -0.113657
6 -0.000918 1.921987 0.611785
6 0.000802 -1.575753 0.616912
6 1.772603 -0.110735 -0.536742
6 -1.772579 -0.112473 -0.536181
8 2.844003 -0.279314 -0.927767

8 -0.001164 2.939425 1.128818
8 0.001325 -2.600046 1.152986
8 -2.843937 -0.282084 -0.926873
1 0.000203 -0.543029 -1.510547

Frequencies, cm^{-1}

-477.0812 57.7446 71.4861
100.3468 101.5870 111.5049
315.1969 344.6206 357.1282
384.2843 454.6197 455.9880
496.5210 545.2396 578.0036
600.5152 626.7145 748.0606
783.2301 1934.7896 2059.4974
2073.7820 2118.6125 2178.4315

$HCo(CO)_4$ molecule

Quasi-TBP

Charge = 0 Multiplicity = 1

Cartesian Coordinates

1 0.003304 0.000680 -1.668185
27 0.000311 -0.000212 -0.189526
6 -0.004400 -0.001692 1.617035
6 1.419121 -1.071382 -0.459149
6 -1.636412 -0.691150 -0.466068
6 0.221166 1.763710 -0.459780
8 -0.007601 -0.002729 2.762434
8 0.361127 2.878838 -0.692820
8 2.316674 -1.747777 -0.692191
8 -2.671267 -1.127317 -0.703276

Frequencies, cm^{-1}

66.9070 67.2561 96.0268
96.2251 106.7835 338.5069
338.5622 349.8822 417.1907
423.4022 423.9839 466.2012
506.1333 506.5025 559.4851
571.2784 571.4117 773.2286
773.7366 1997.0483 2121.1796
2121.3877 2142.4476 2192.2420

TS_{BPR}

Charge = 0 Multiplicity = 1

Cartesian Coordinates

1 -0.052579 -0.023835 1.672708
27 -0.004769 -0.002225 0.153290
6 -1.780776 0.010118 -0.120869
6 0.010338 1.784384 -0.039797
6 -0.006286 -1.782719 -0.090316
6 1.784934 -0.008196 -0.008961
8 -2.923852 0.017121 -0.224602
8 2.932278 -0.012889 -0.040747
8 0.017621 2.930987 -0.091622
8 -0.009537 -2.927420 -0.174512

Frequencies, cm^{-1}

-192.1987 101.6475 102.2351
102.2511 116.3070 178.7146
357.8278 357.8331 370.4313
421.8593 440.7414 462.2406
462.2576 552.5110 616.0508
616.0684 628.0113 655.4265
655.4375 1763.7055 2117.2016
2117.2233 2122.7036 2188.8293

TS_{OSH}

Charge = 0 Multiplicity = 1

Cartesian Coordinates
1 0.000120 -0.000755 -1.686898
27 0.000027 -0.000085 -0.189329
6 -1.694457 -0.000545 -0.711506
6 -0.000350 1.513947 0.815133
6 0.000049 -1.513211 0.816503
6 1.694801 -0.000099 -0.710870
8 -2.776183 -0.000861 -1.097411
8 2.776665 -0.000131 -1.096368
8 -0.000637 2.474604 1.442220
8 0.000015 -2.473299 1.444461

Frequencies, cm^{-1}

-322.7462 67.4825 85.3093
97.7075 108.1201 110.9899 329.4634 349.0703
371.6325
412.9894 415.8638 470.1476
486.9389 496.1336 526.0941
549.0666 583.1324 618.9726
636.0568 1885.6404 2121.9183
2122.6343 2129.8936 2186.9533
